# Supplementary figures and images for: GFPT2/GFAT2 and AMDHD2 act in tandem to control the hexosamine pathway
Source: eLife. 2022 Mar 1;11:e69223. doi: 10.7554/eLife.69223 (PMC8970586; doi:10.7554/eLife.69223)

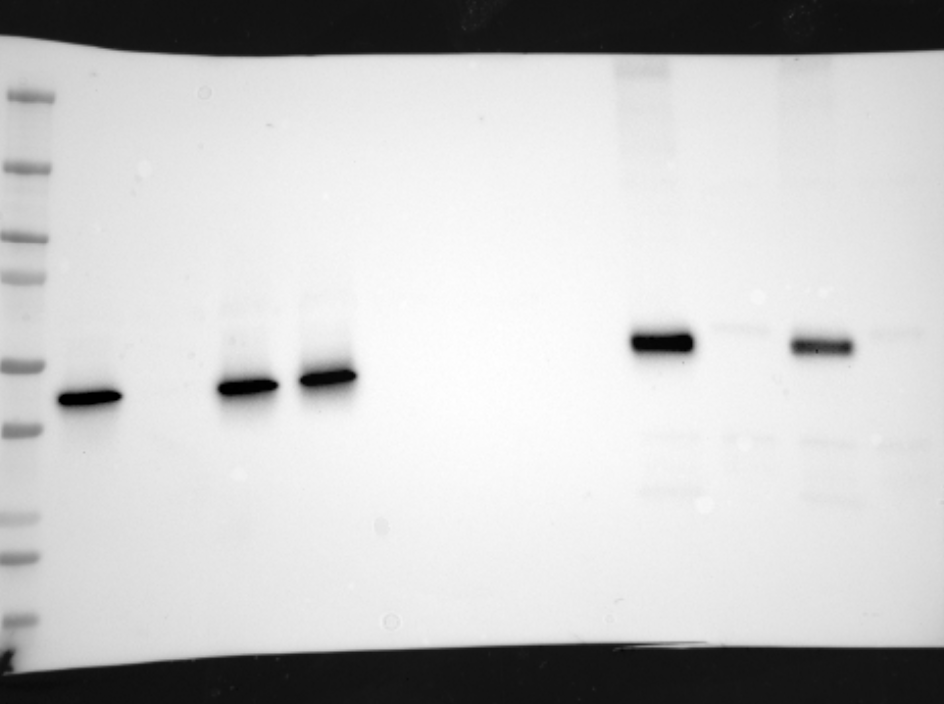

Supplement: Figure 1—source data 1. [file elife-69223-fig1-data1.zip › Figure 1/WesternBlots/Figure1D-1-full-raw-unedited.tif]

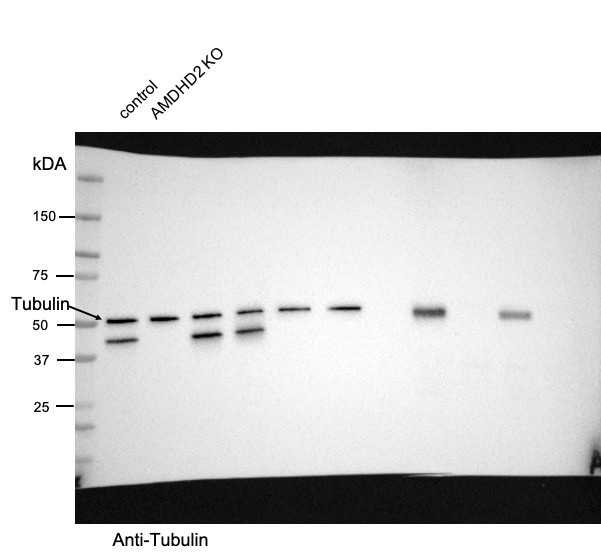

Supplement: Figure 1—source data 1. [file elife-69223-fig1-data1.zip › Figure 1/WesternBlots/Figure1D-2-labeled.jpg]

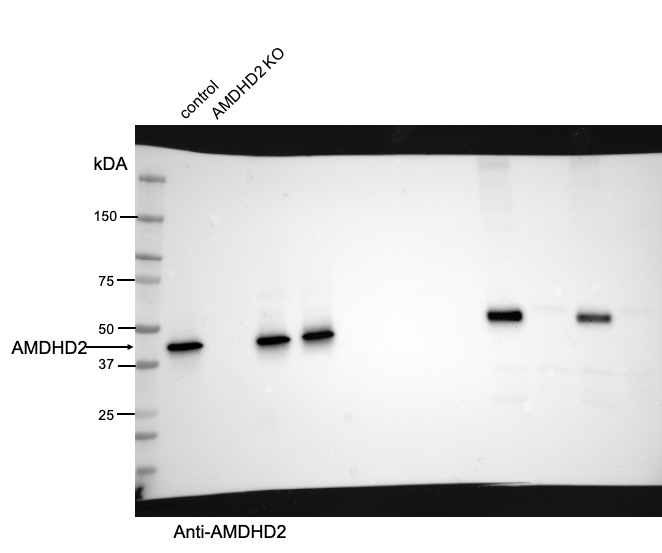

Supplement: Figure 1—source data 1. [file elife-69223-fig1-data1.zip › Figure 1/WesternBlots/Figure1D-1-labeled.jpg]

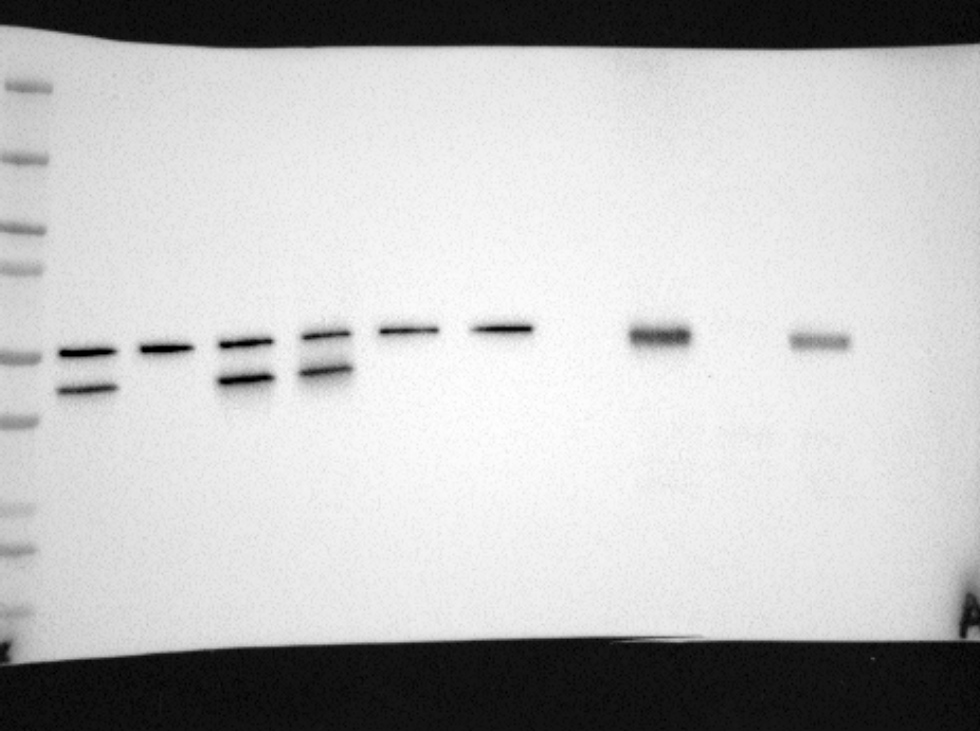

Supplement: Figure 1—source data 1. [file elife-69223-fig1-data1.zip › Figure 1/WesternBlots/Figure1D-2-full-raw-unedited.tif]

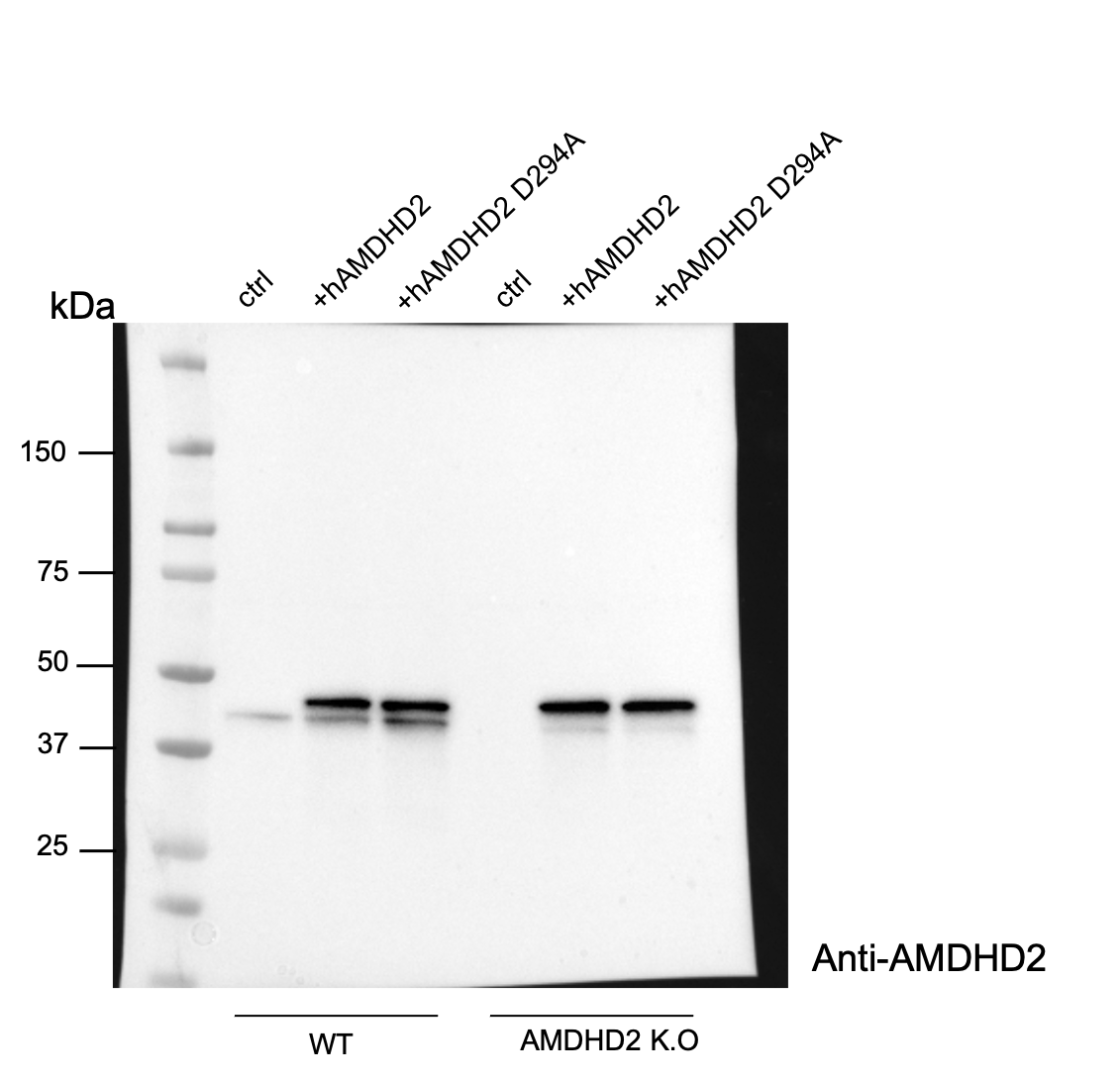

Supplement: Figure 2—figure supplement 2—source data 1. [file elife-69223-fig2-figsupp2-data1.zip › Figure 2-figure supplement 2/WesternBlots/Figure2FigureSupplement1A-1-labeled.png]

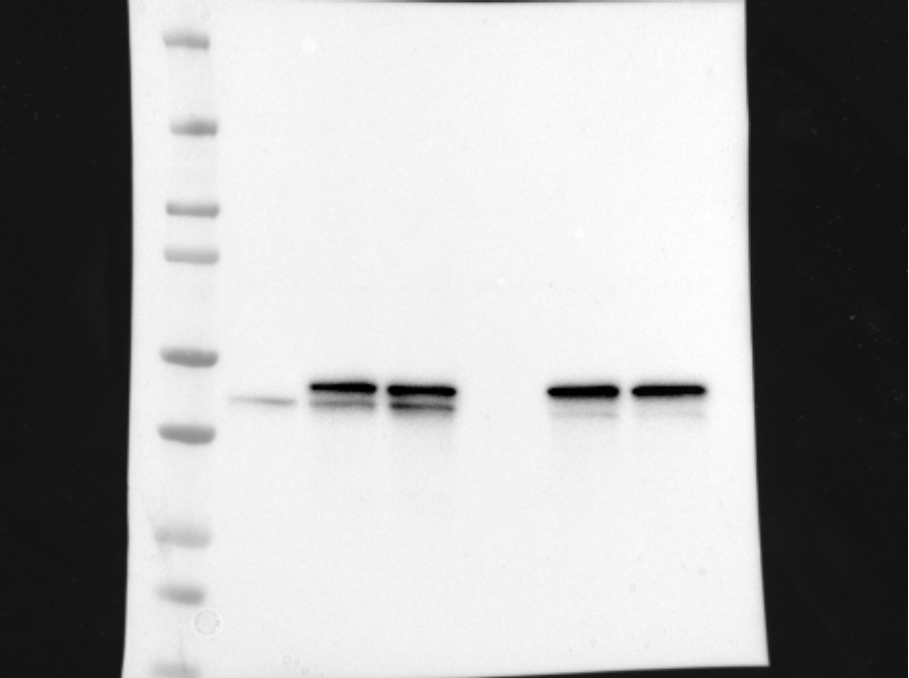

Supplement: Figure 2—figure supplement 2—source data 1. [file elife-69223-fig2-figsupp2-data1.zip › Figure 2-figure supplement 2/WesternBlots/Figure2FigureSupplement1A-1-full-raw-unedited.tif]

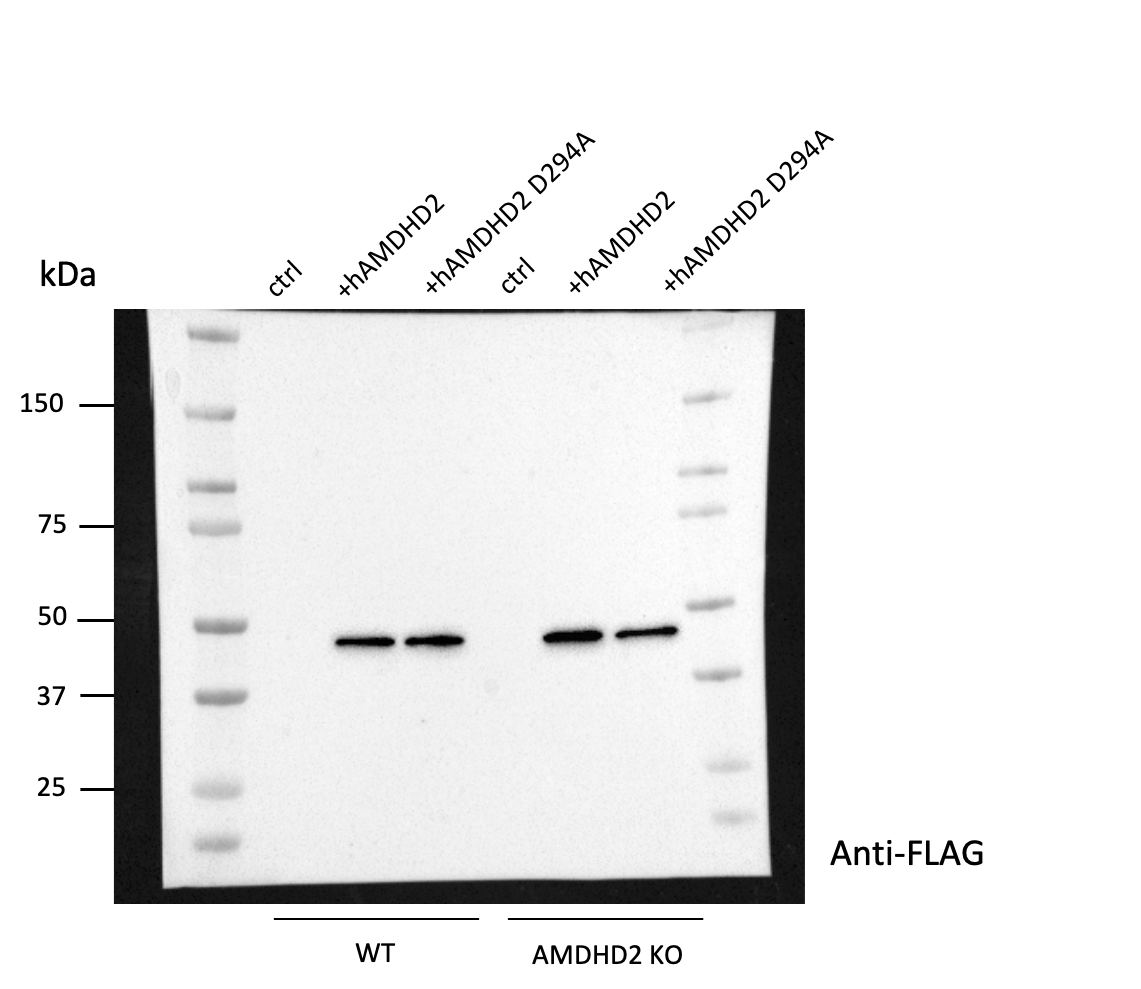

Supplement: Figure 2—figure supplement 2—source data 1. [file elife-69223-fig2-figsupp2-data1.zip › Figure 2-figure supplement 2/WesternBlots/Figure2FigureSupplement1B-1-labeled.png]

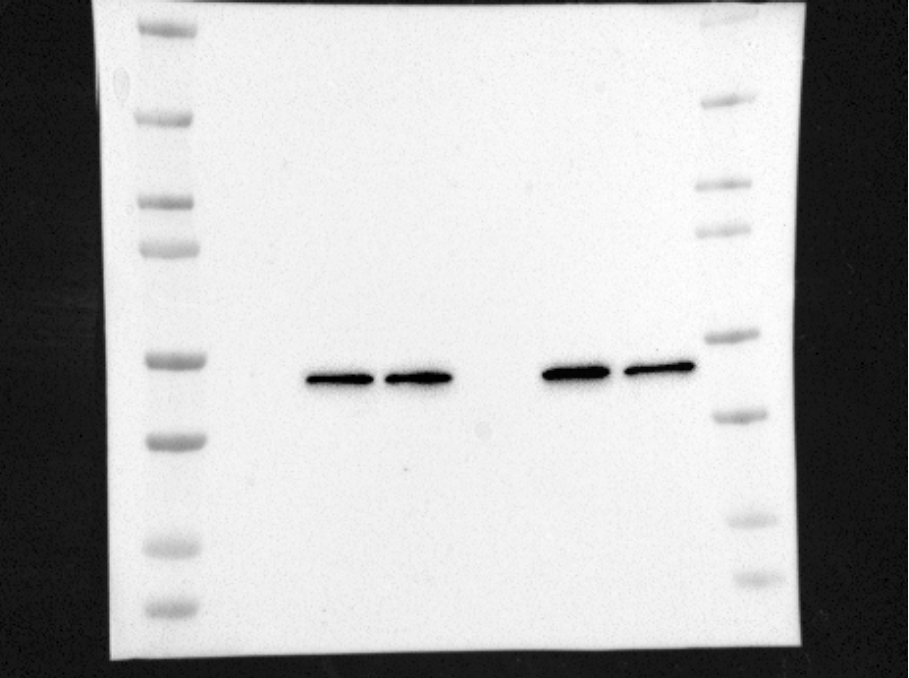

Supplement: Figure 2—figure supplement 2—source data 1. [file elife-69223-fig2-figsupp2-data1.zip › Figure 2-figure supplement 2/WesternBlots/Figure2FigureSupplement1B-1-full-raw-unedited.tif]

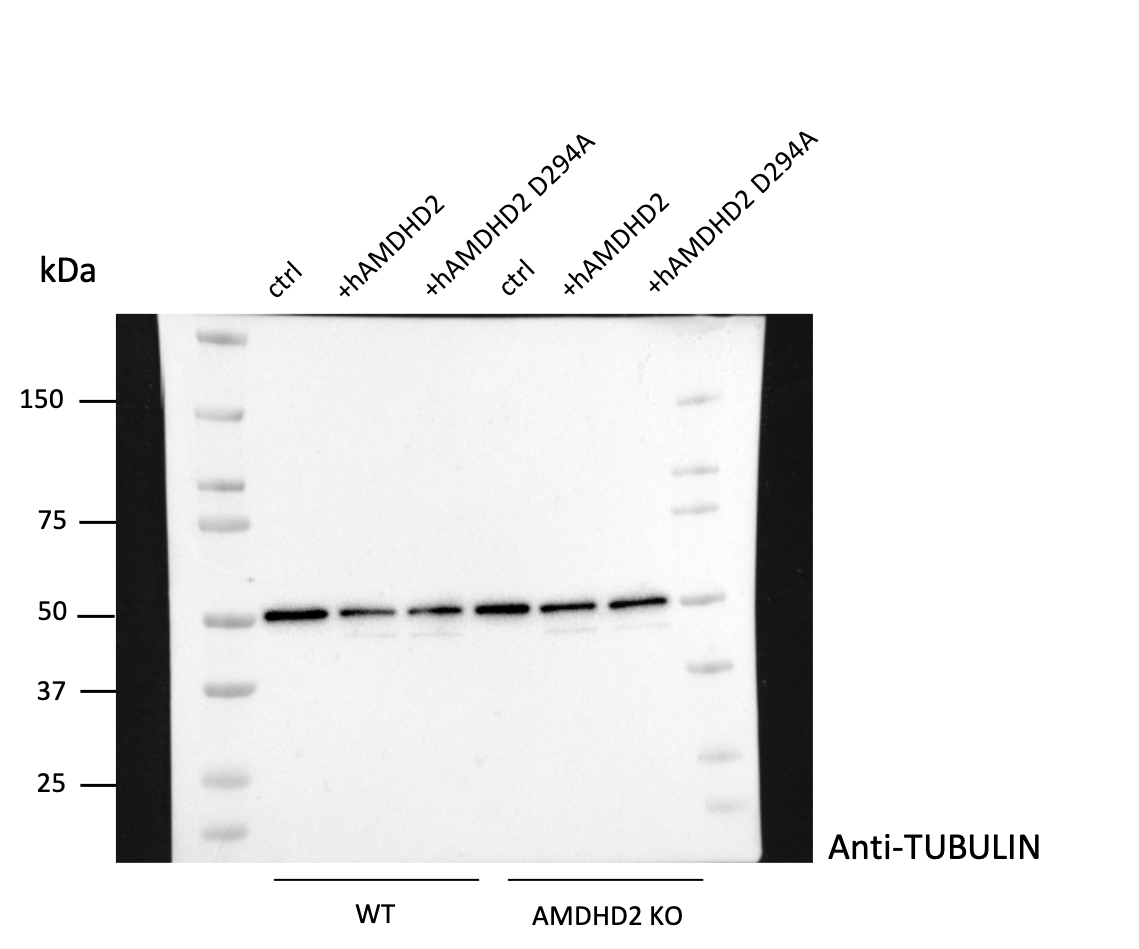

Supplement: Figure 2—figure supplement 2—source data 1. [file elife-69223-fig2-figsupp2-data1.zip › Figure 2-figure supplement 2/WesternBlots/Figure2FigureSupplement1B-2-labeled.png]

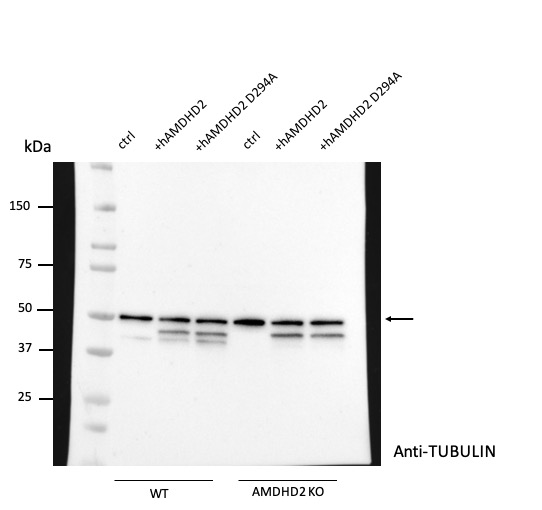

Supplement: Figure 2—figure supplement 2—source data 1. [file elife-69223-fig2-figsupp2-data1.zip › Figure 2-figure supplement 2/WesternBlots/Figure2FigureSupplement1A-2-labeled.jpg]

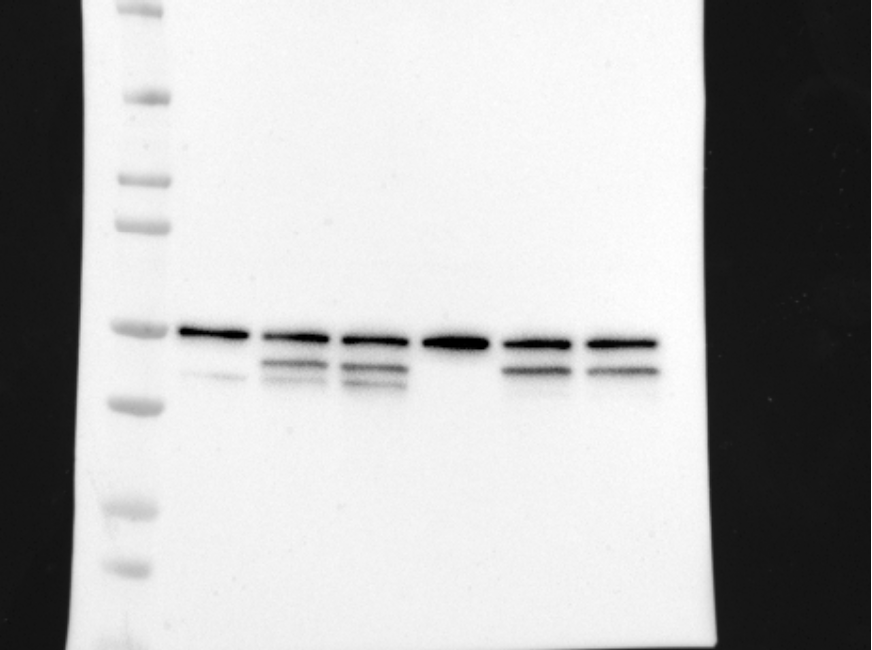

Supplement: Figure 2—figure supplement 2—source data 1. [file elife-69223-fig2-figsupp2-data1.zip › Figure 2-figure supplement 2/WesternBlots/Figure2FigureSupplement1A-2-full-raw-unedited.tif]

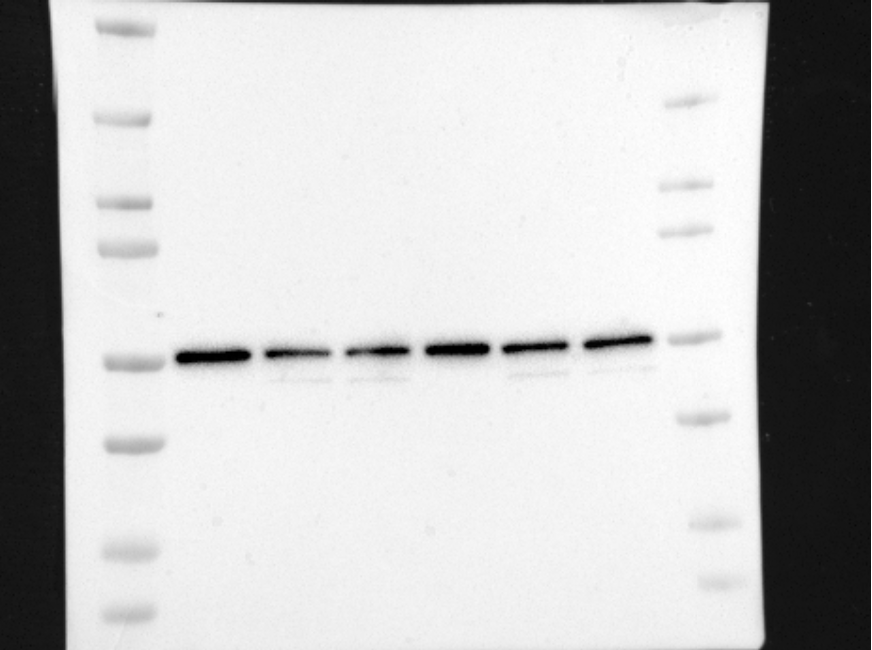

Supplement: Figure 2—figure supplement 2—source data 1. [file elife-69223-fig2-figsupp2-data1.zip › Figure 2-figure supplement 2/WesternBlots/Figure2FigureSupplement1B-2-full-raw-unedited.tif]

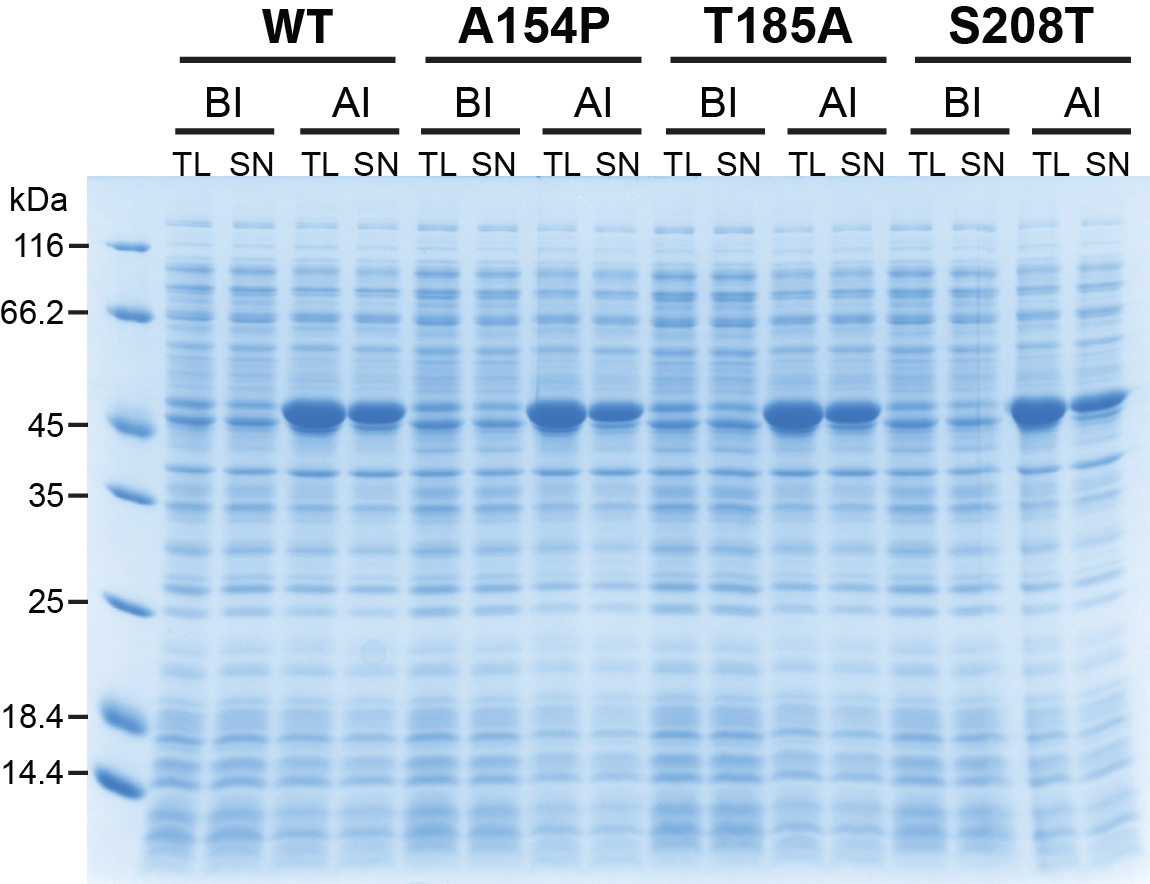

Supplement: Figure 4—source data 1. [file elife-69223-fig4-data1.zip › Figure 4/Figure4a-3-labeled.jpg]

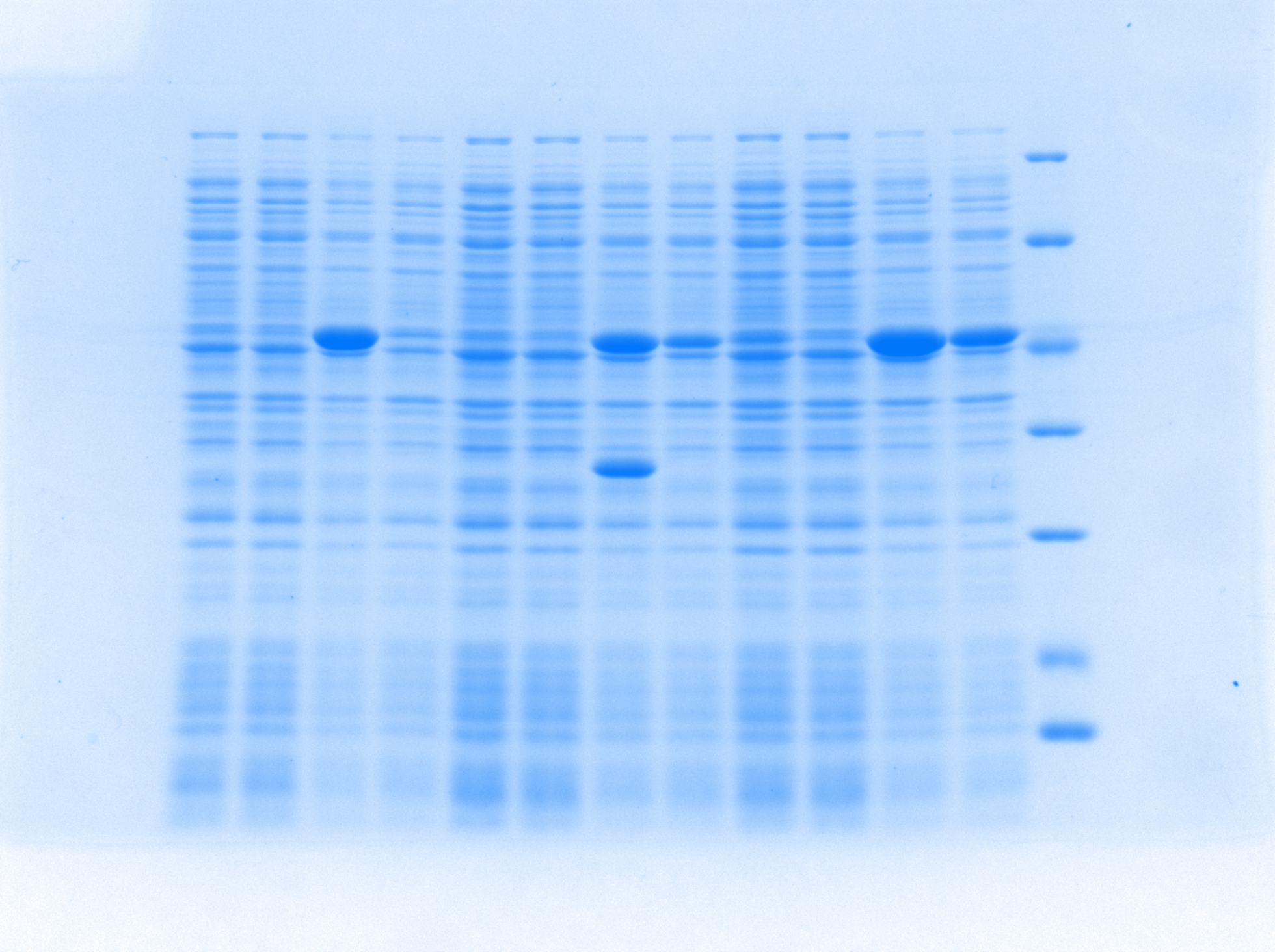

Supplement: Figure 4—source data 1. [file elife-69223-fig4-data1.zip › Figure 4/Figure4a-2-full-raw-unedited.jpg]

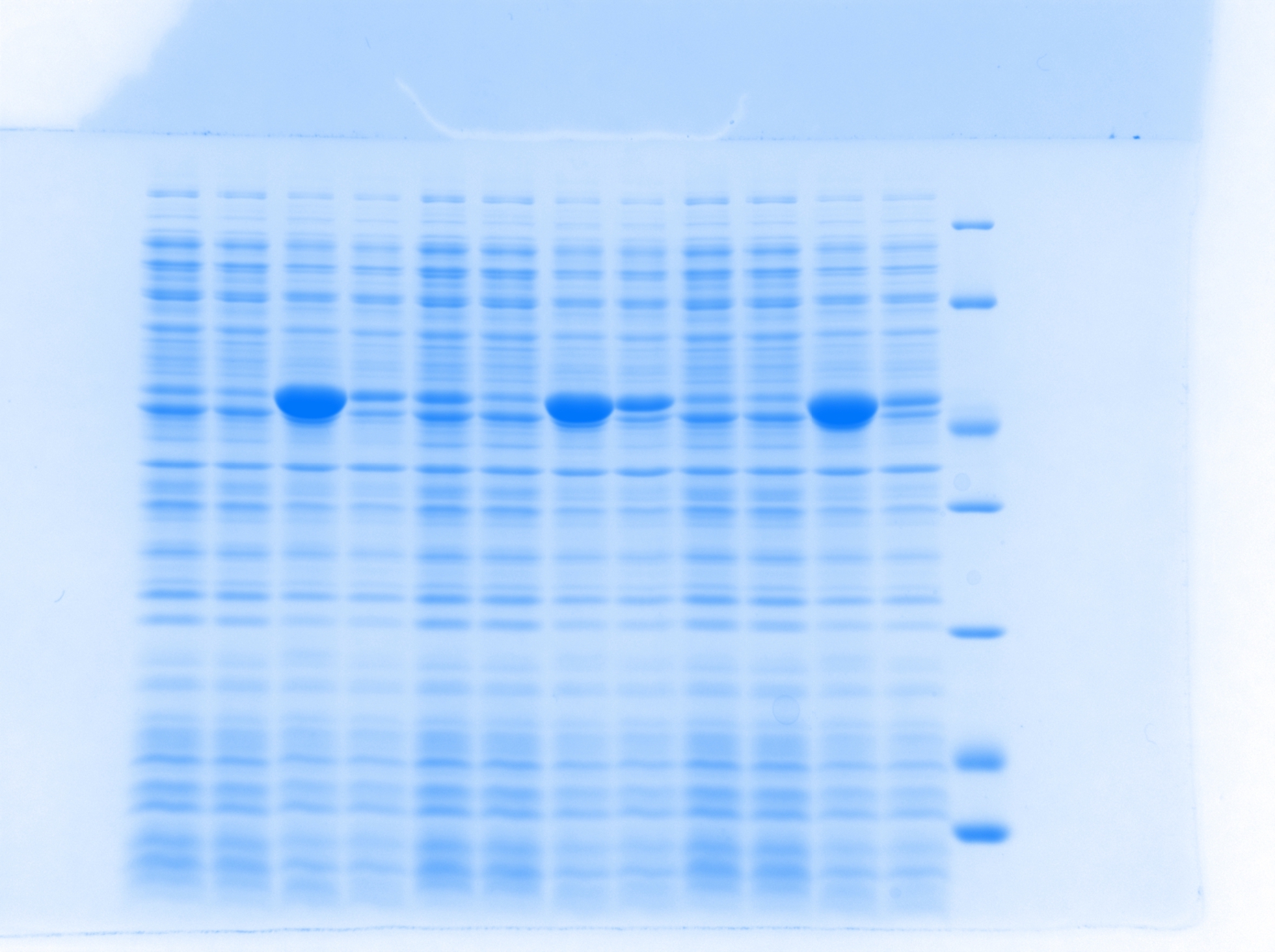

Supplement: Figure 4—source data 1. [file elife-69223-fig4-data1.zip › Figure 4/Figure4a-4-full-raw-unedited.jpg]

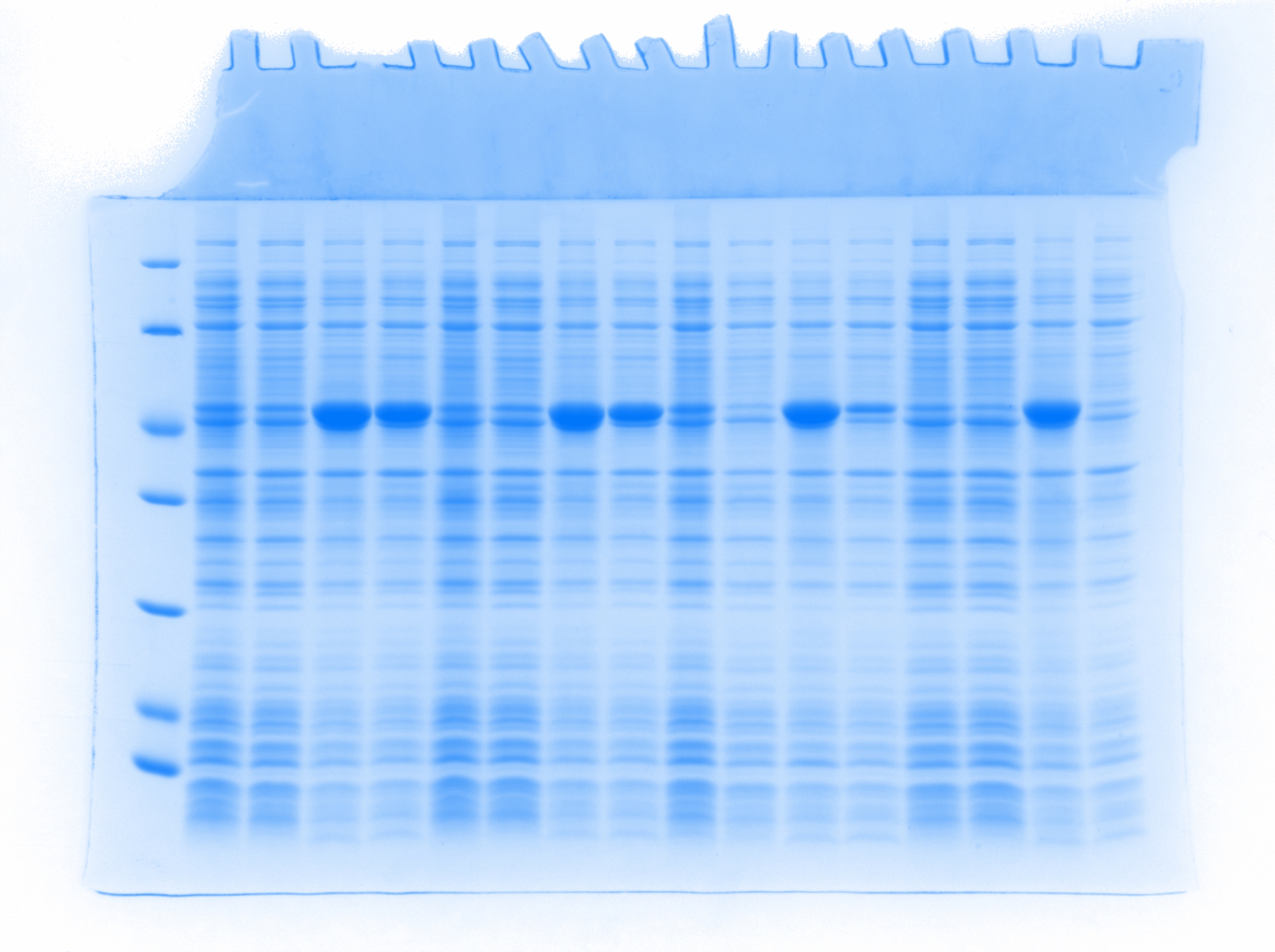

Supplement: Figure 4—source data 1. [file elife-69223-fig4-data1.zip › Figure 4/Figure4a-1-full-raw-unedited.jpg]

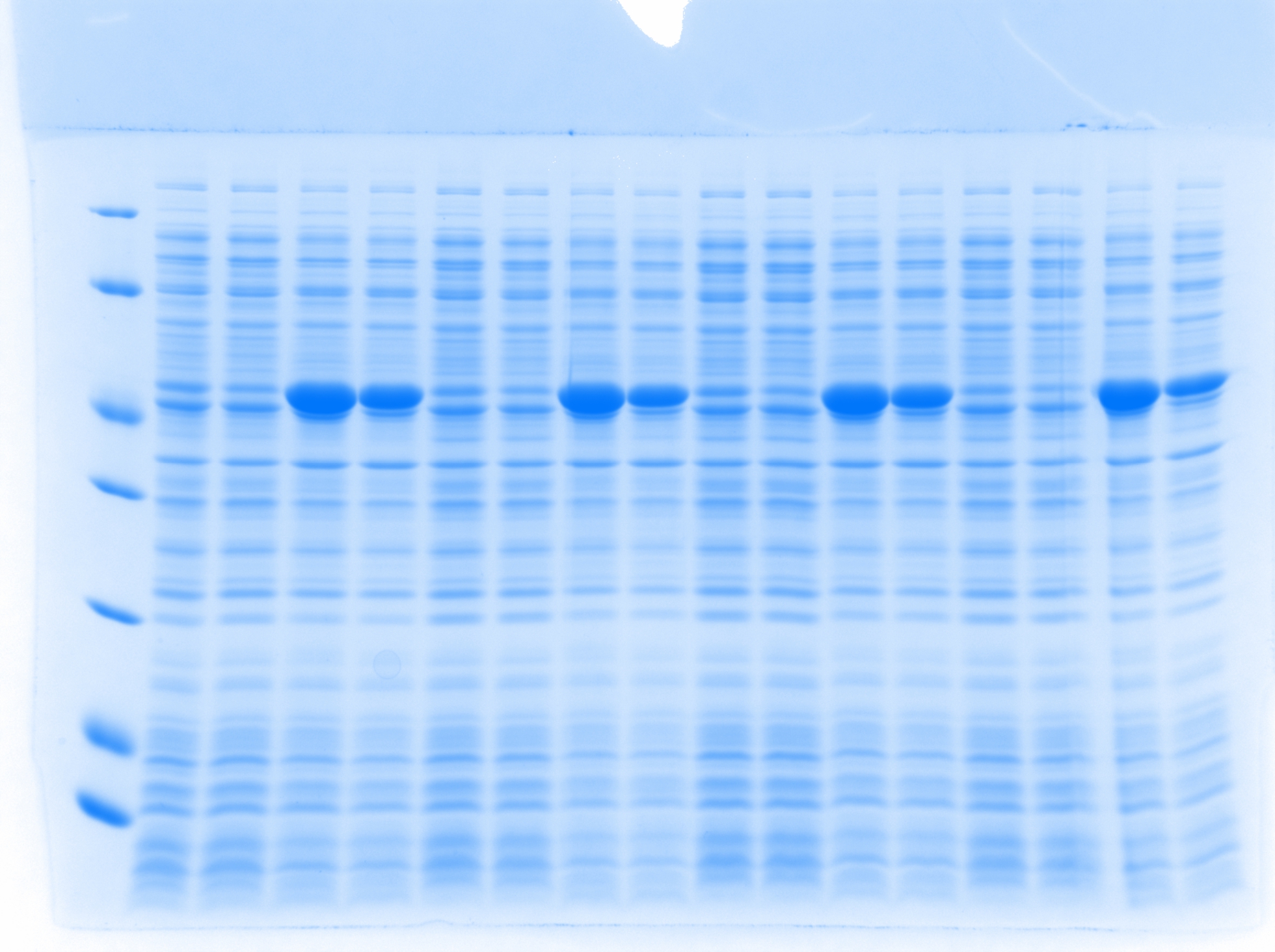

Supplement: Figure 4—source data 1. [file elife-69223-fig4-data1.zip › Figure 4/Figure4a-3-full-raw-unedited.jpg]

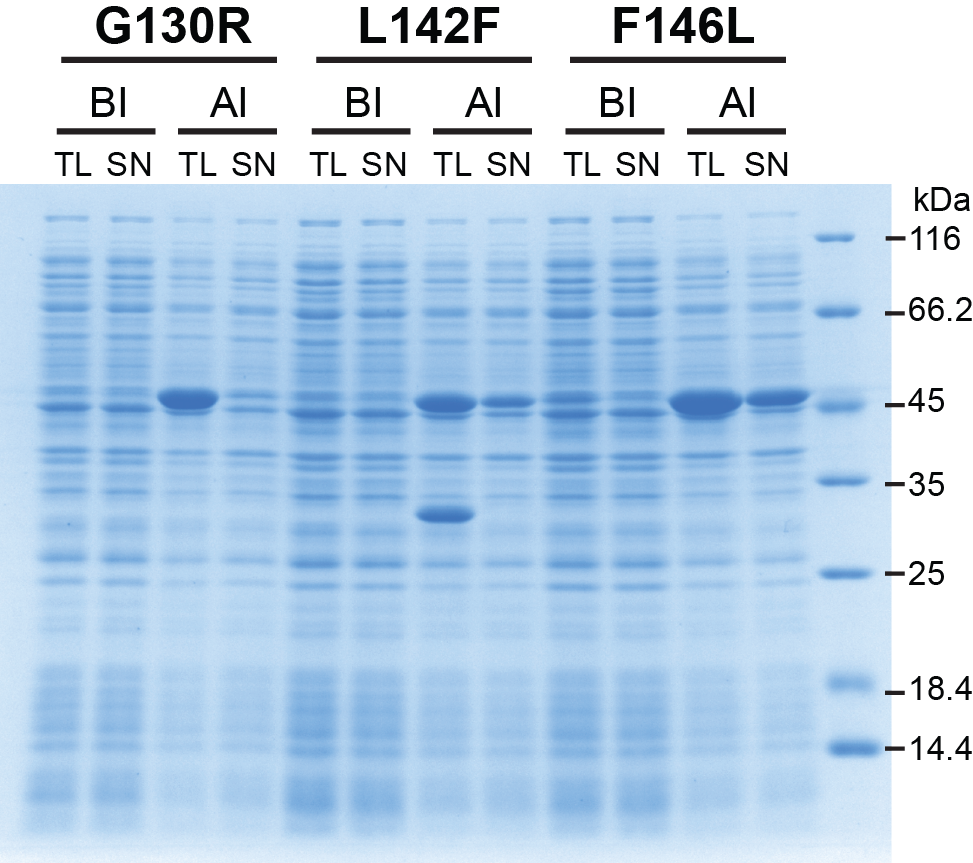

Supplement: Figure 4—source data 1. [file elife-69223-fig4-data1.zip › Figure 4/Figure4a-2-labeled.jpg]

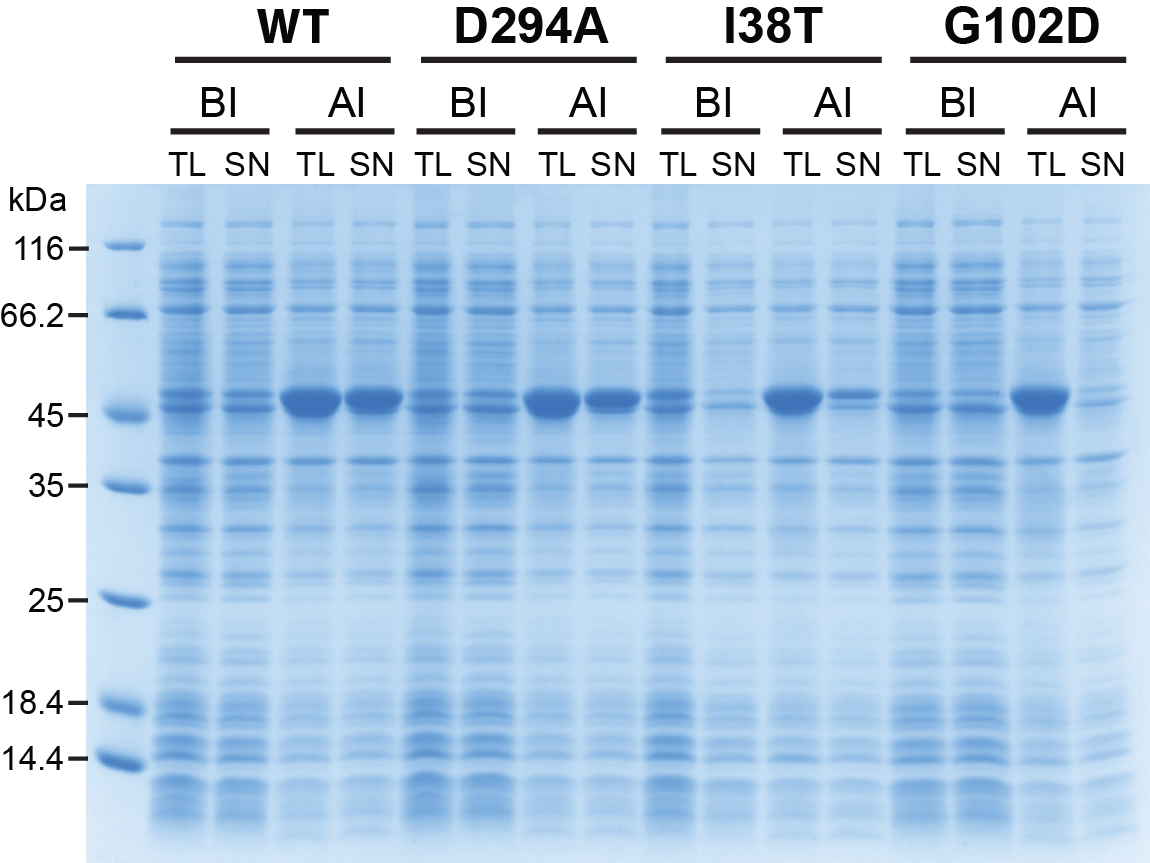

Supplement: Figure 4—source data 1. [file elife-69223-fig4-data1.zip › Figure 4/Figure4a-1-labeled.jpg]

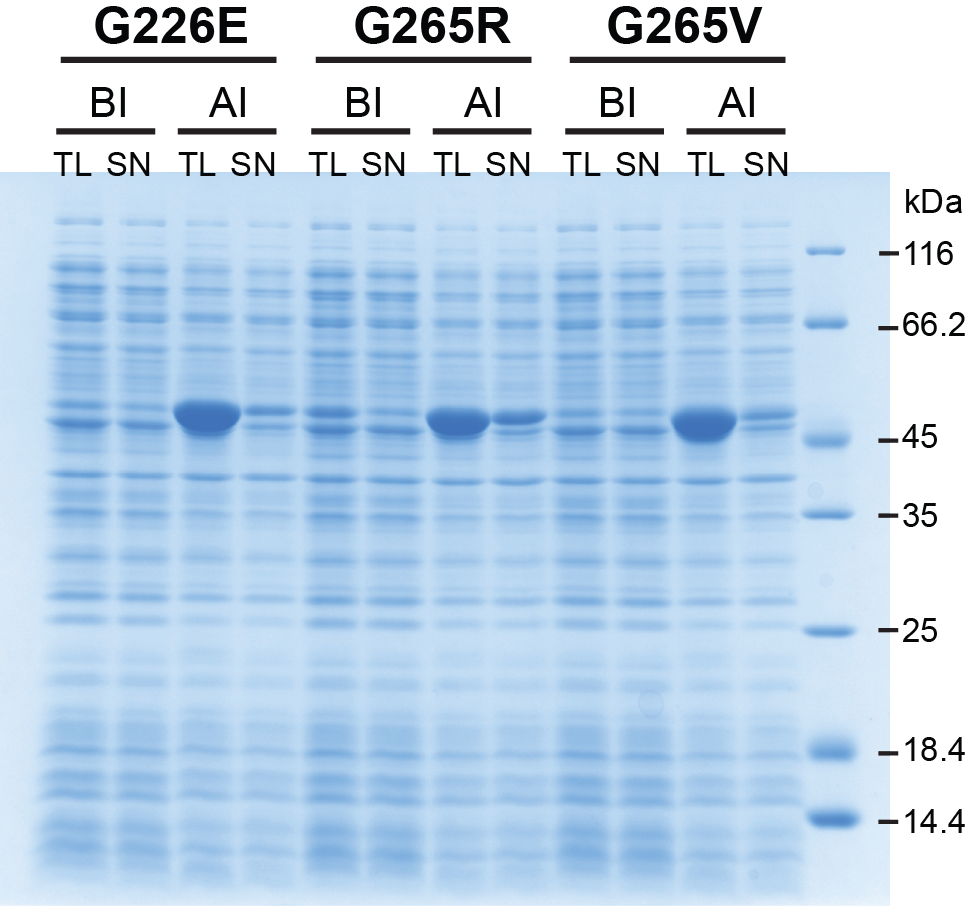

Supplement: Figure 4—source data 1. [file elife-69223-fig4-data1.zip › Figure 4/Figure4a-4-labeled.jpg]

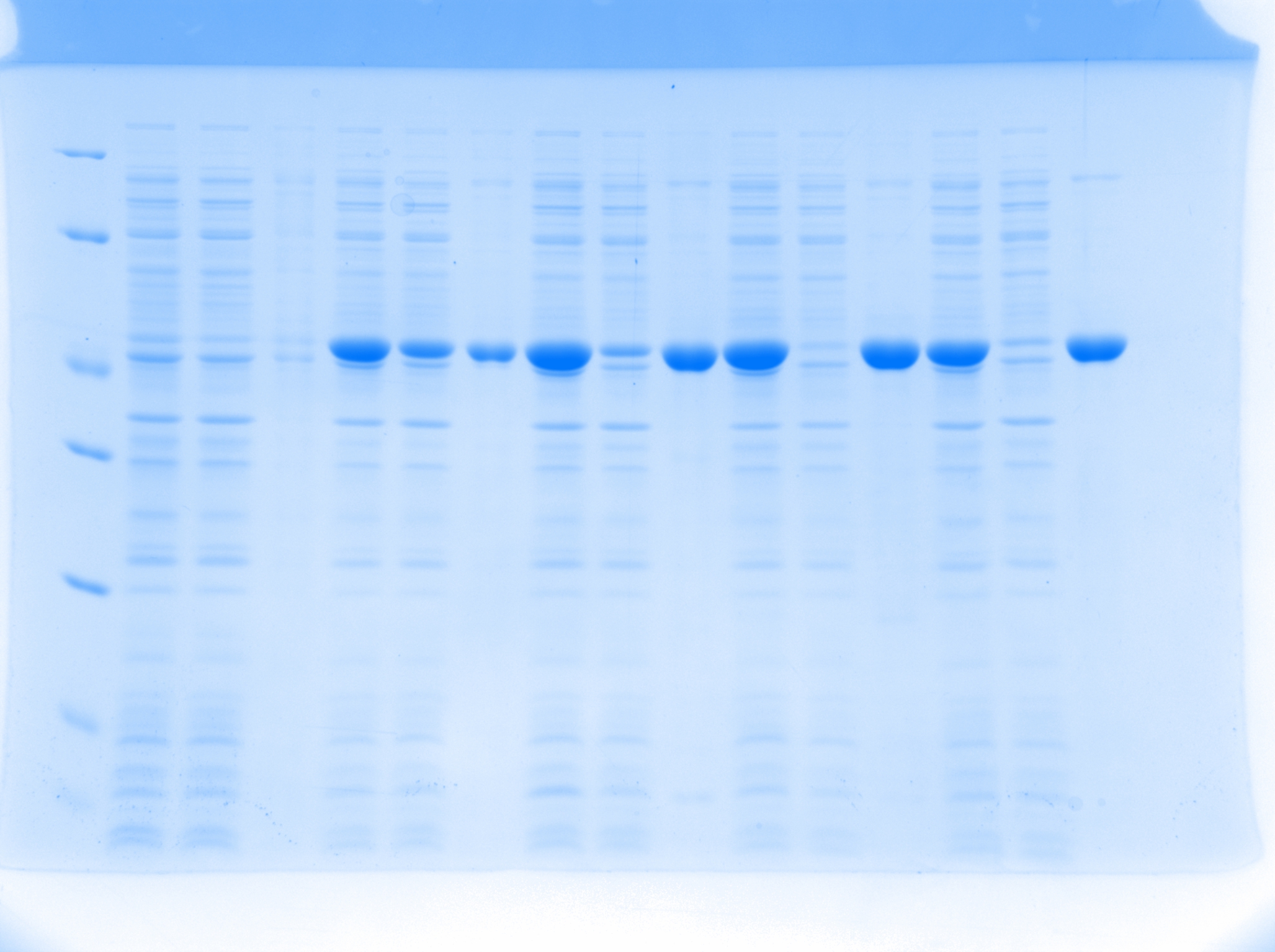

Supplement: Figure 4—figure supplement 1—source data 1. [file elife-69223-fig4-figsupp1-data1.zip › Figure 4-figure supplement 1/Figure4FigureSupplement1-1-full-raw-unedited.jpg]

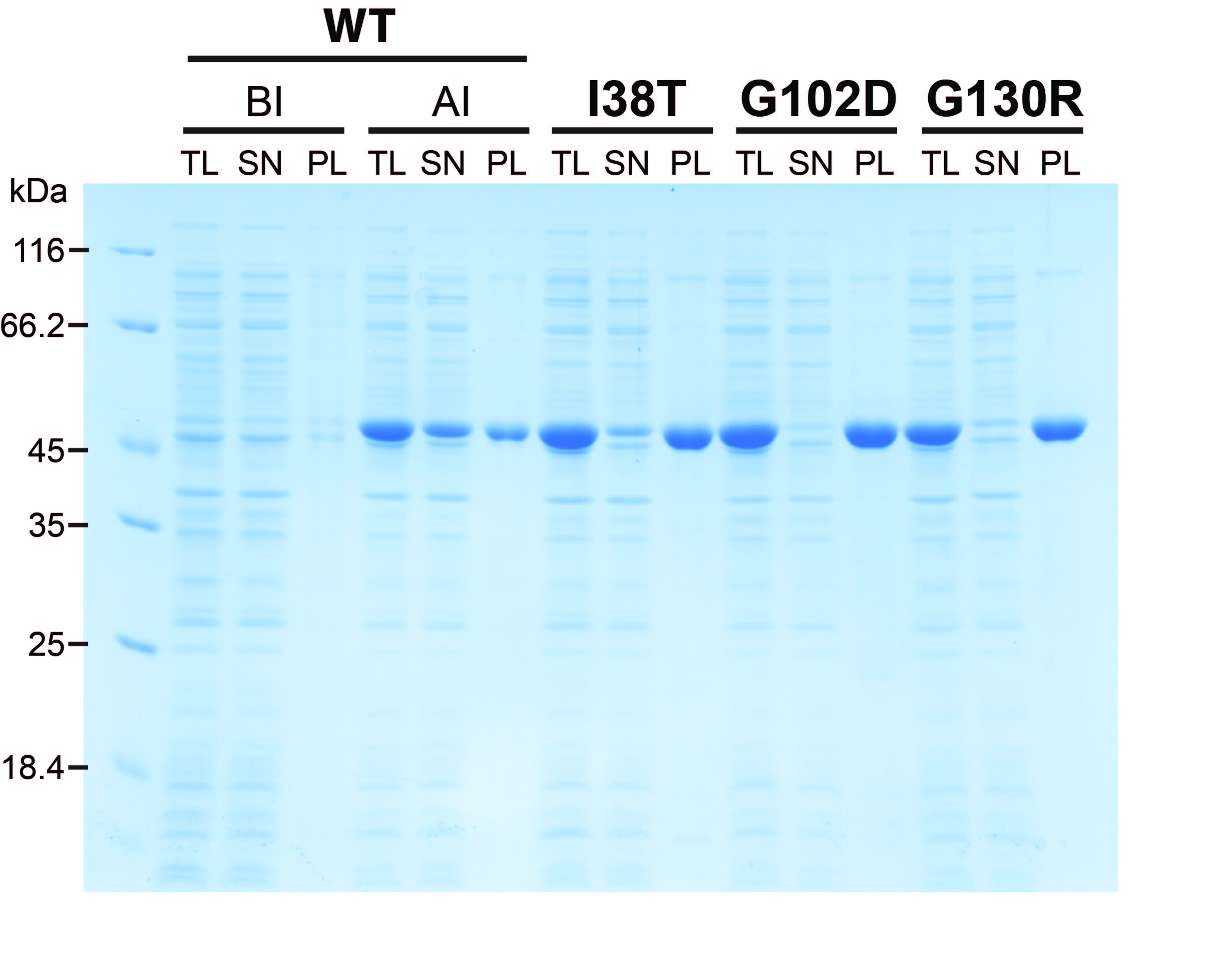

Supplement: Figure 4—figure supplement 1—source data 1. [file elife-69223-fig4-figsupp1-data1.zip › Figure 4-figure supplement 1/Figure4FigureSupplement1-1-labeled.jpg]

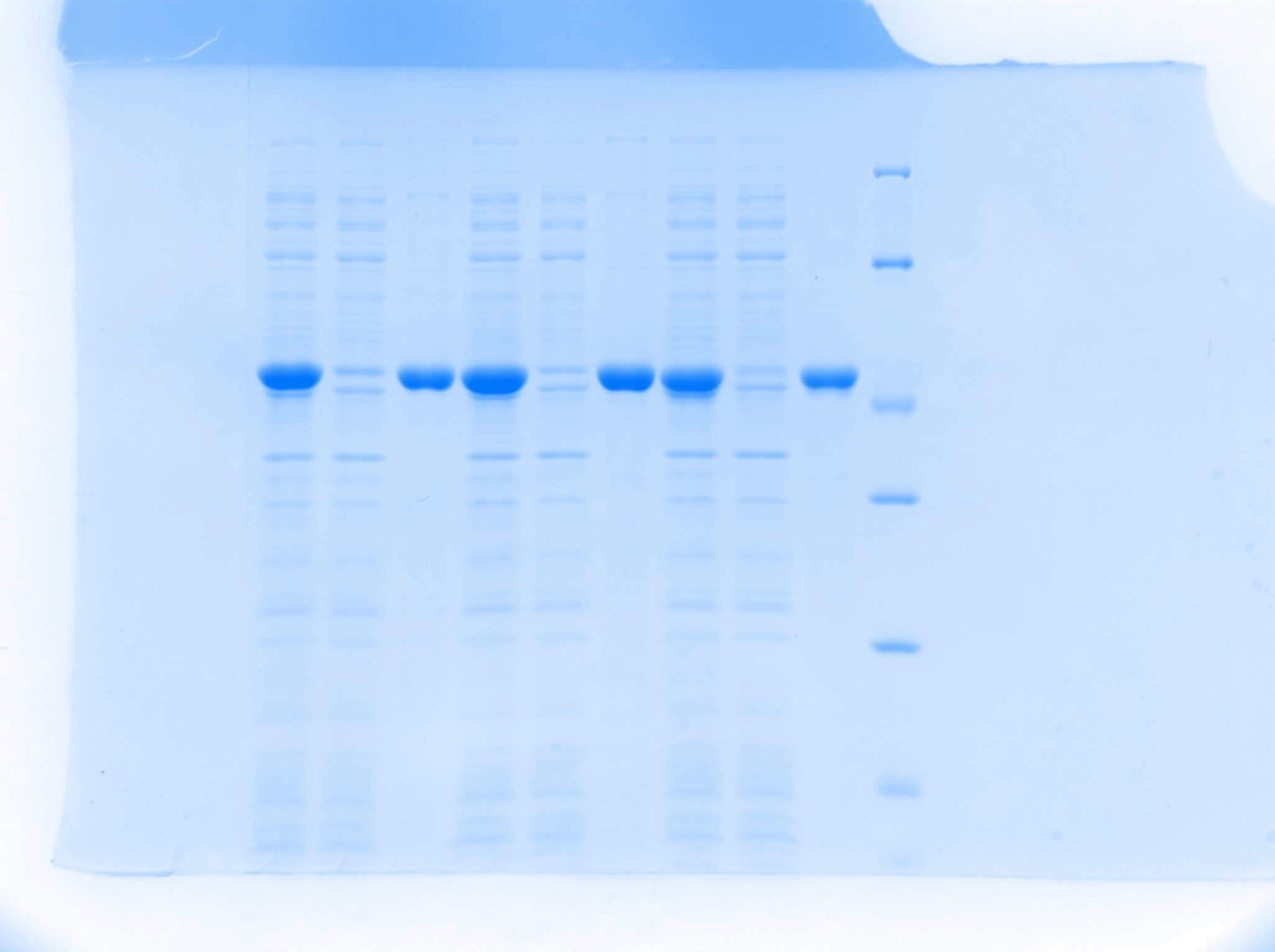

Supplement: Figure 4—figure supplement 1—source data 1. [file elife-69223-fig4-figsupp1-data1.zip › Figure 4-figure supplement 1/Figure4FigureSupplement1-2-full-raw-unedited.jpg]

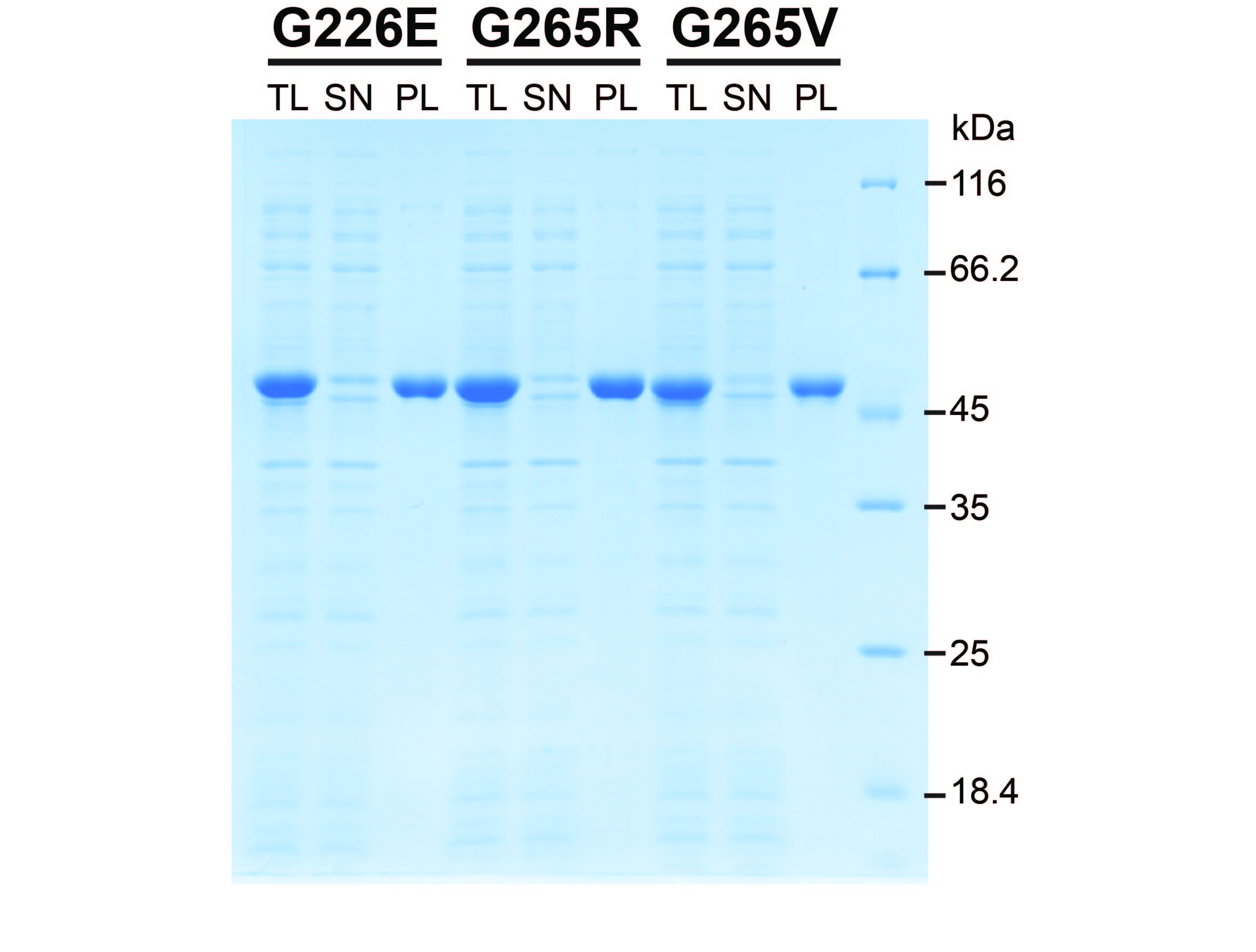

Supplement: Figure 4—figure supplement 1—source data 1. [file elife-69223-fig4-figsupp1-data1.zip › Figure 4-figure supplement 1/Figure4FigureSupplement1-2-labeled.jpg]

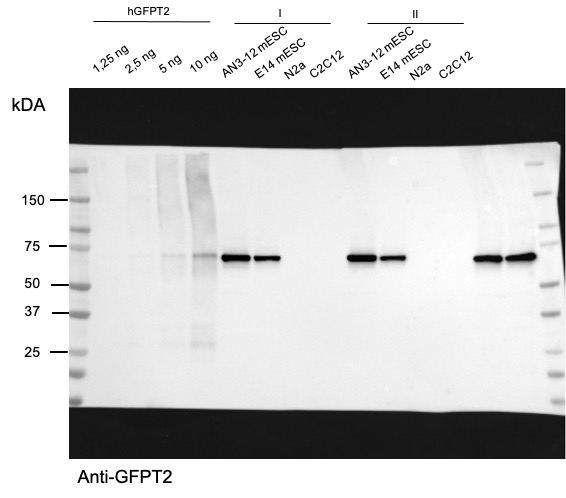

Supplement: Figure 5—source data 1. [file elife-69223-fig5-data1.zip › Figure 5/WesternBlots/Figure5B-3-labeled.jpg]

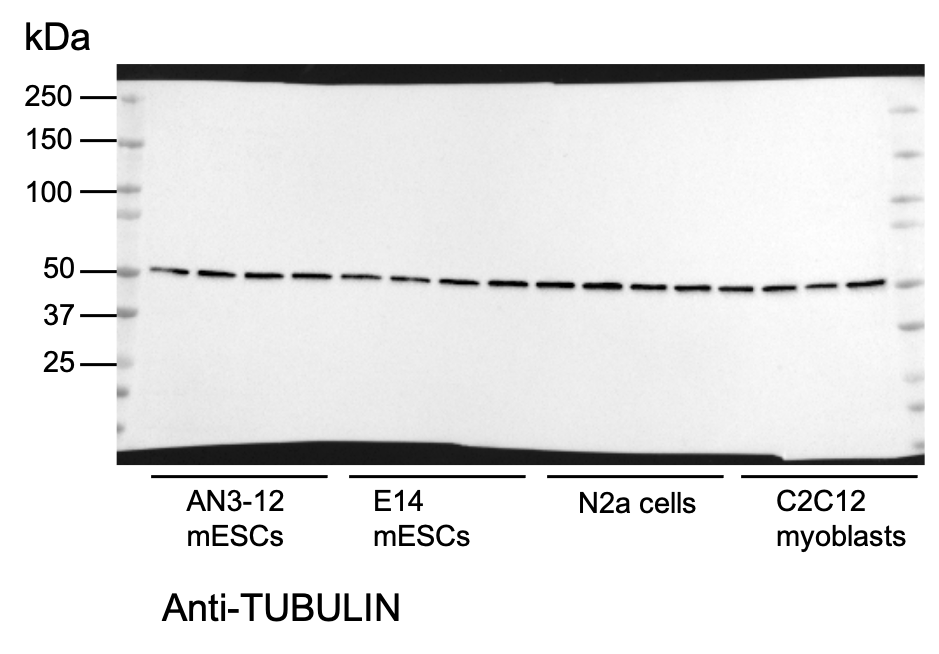

Supplement: Figure 5—source data 1. [file elife-69223-fig5-data1.zip › Figure 5/WesternBlots/Figure5G-4-labeled.png]

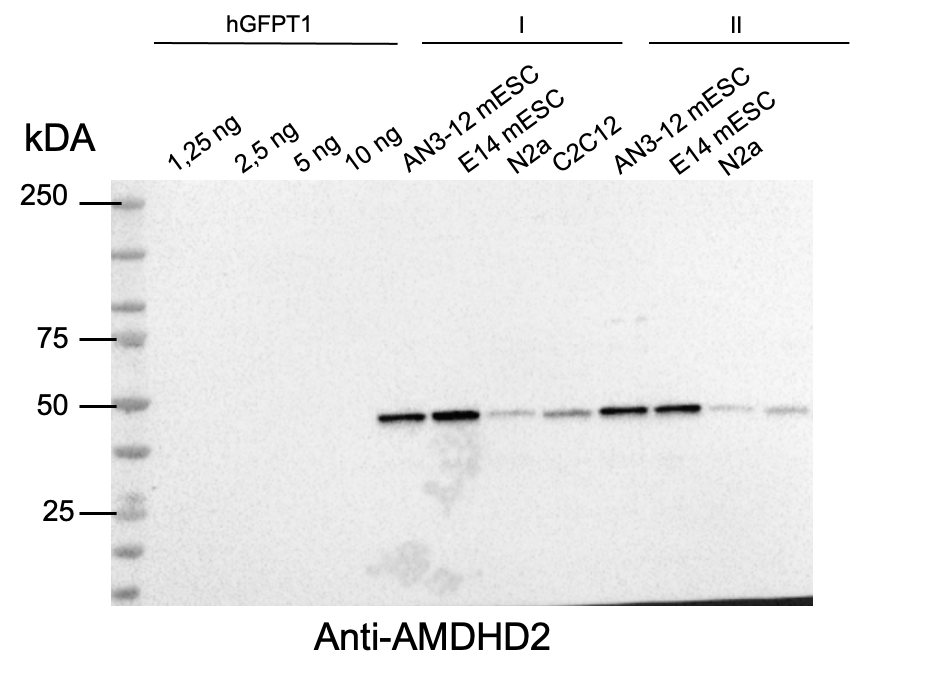

Supplement: Figure 5—source data 1. [file elife-69223-fig5-data1.zip › Figure 5/WesternBlots/Figure5C-1-labeled.png]

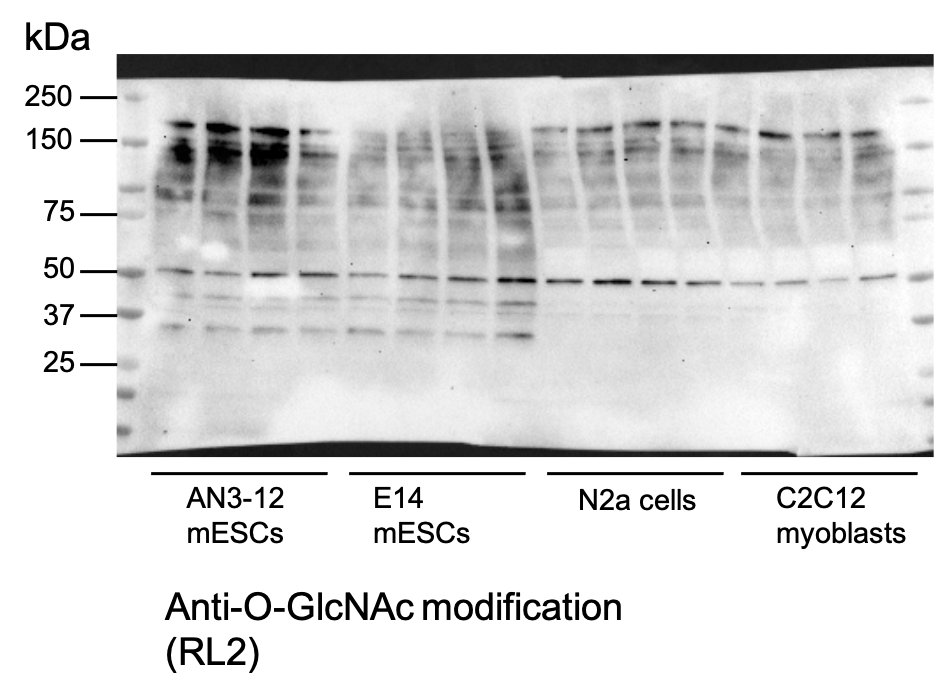

Supplement: Figure 5—source data 1. [file elife-69223-fig5-data1.zip › Figure 5/WesternBlots/Figure5G-1-labeled.png]

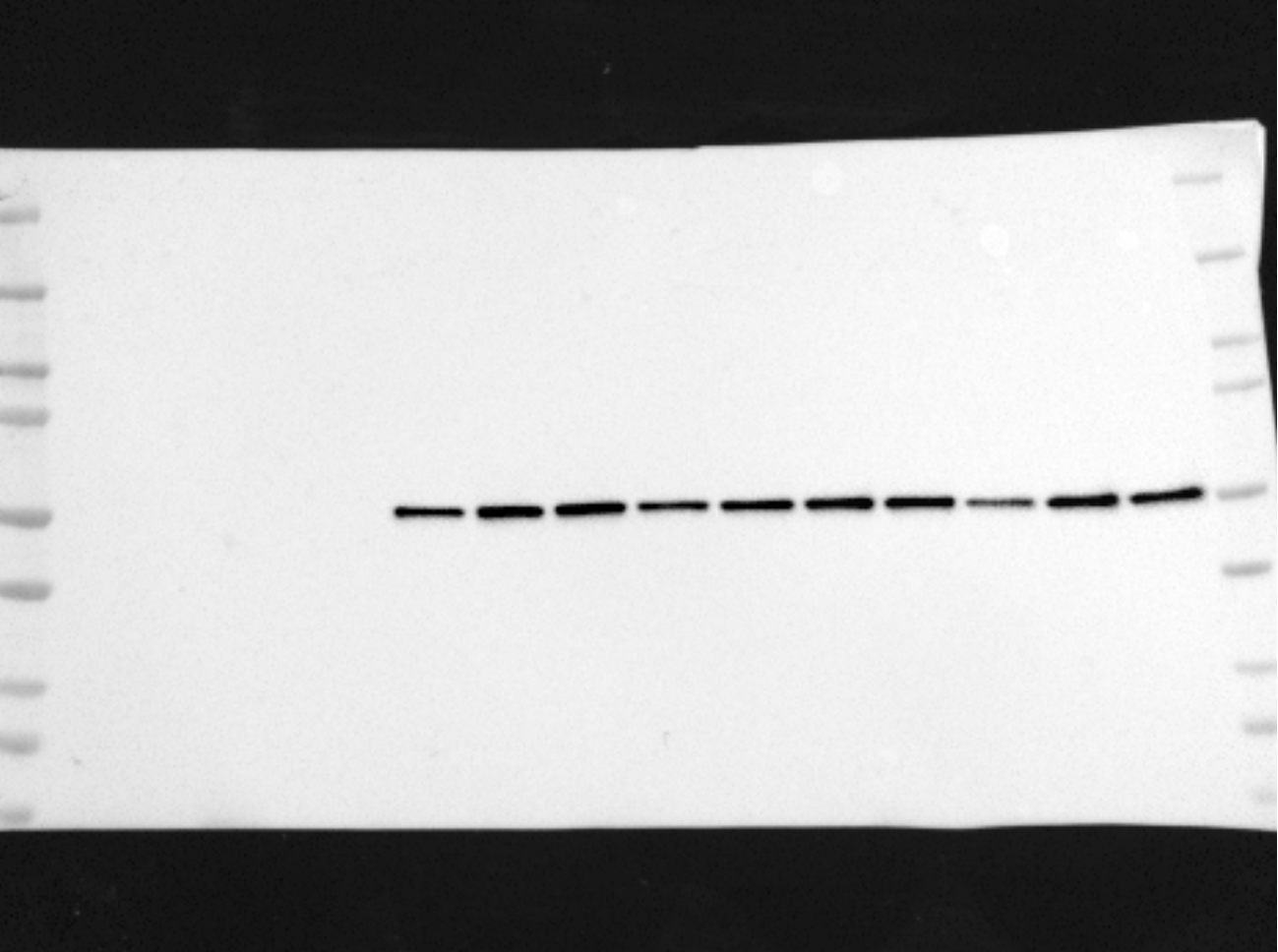

Supplement: Figure 5—source data 1. [file elife-69223-fig5-data1.zip › Figure 5/WesternBlots/Figure5B-4-full-raw-unedited.tif]

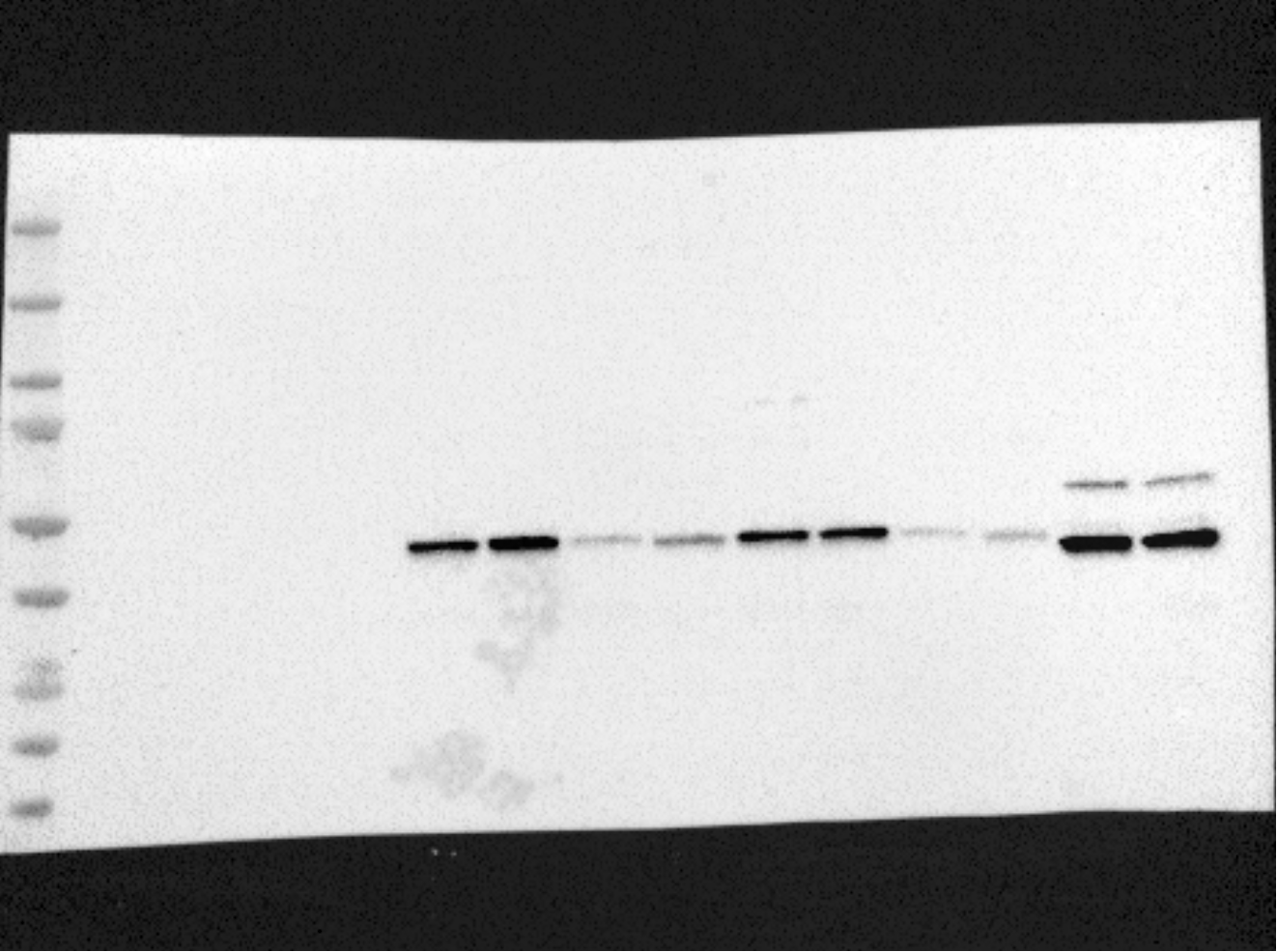

Supplement: Figure 5—source data 1. [file elife-69223-fig5-data1.zip › Figure 5/WesternBlots/Figure5C-1-full-raw-unedited.tif]

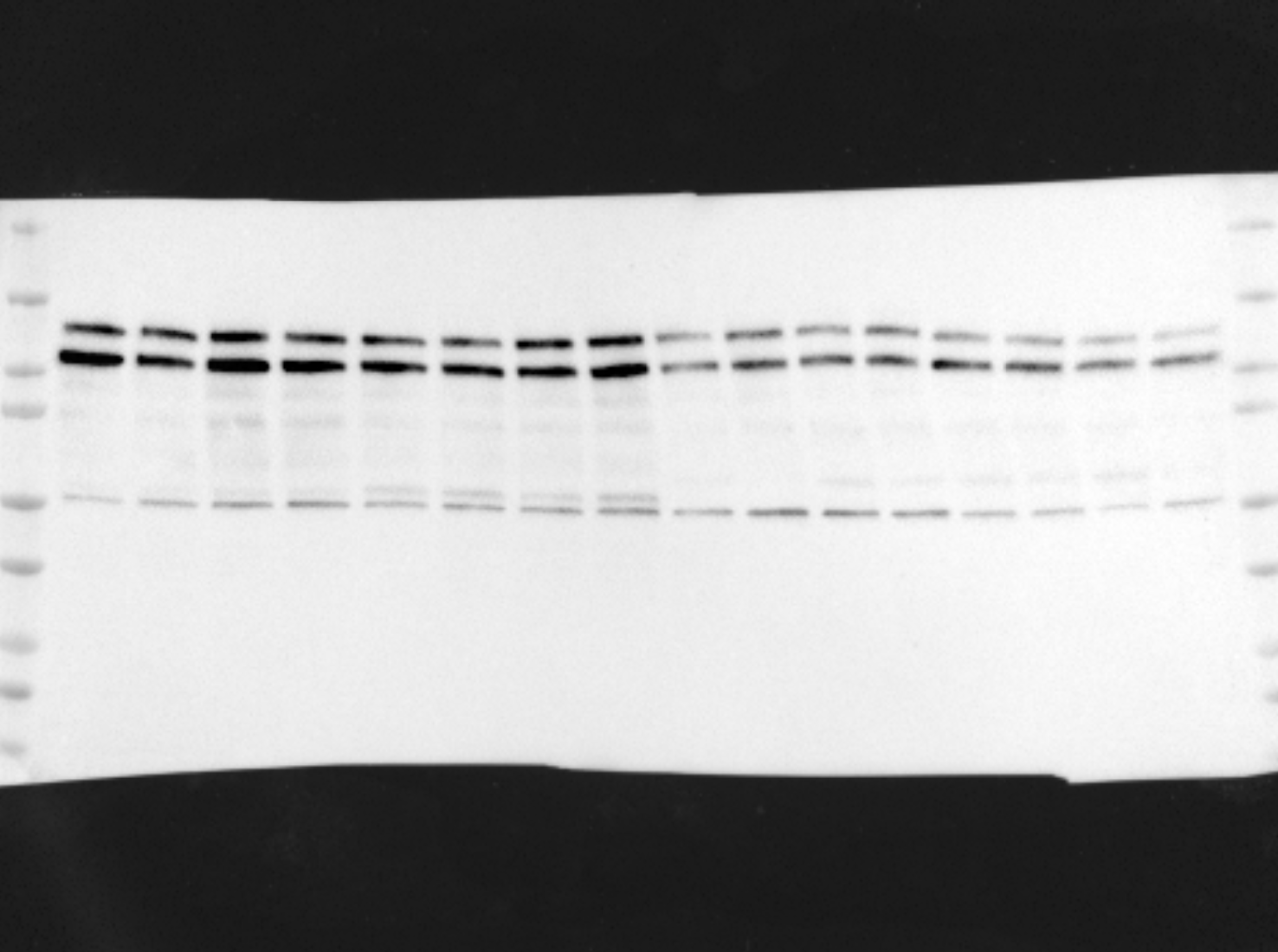

Supplement: Figure 5—source data 1. [file elife-69223-fig5-data1.zip › Figure 5/WesternBlots/Figure5G-3-full-raw-unedited.tif]

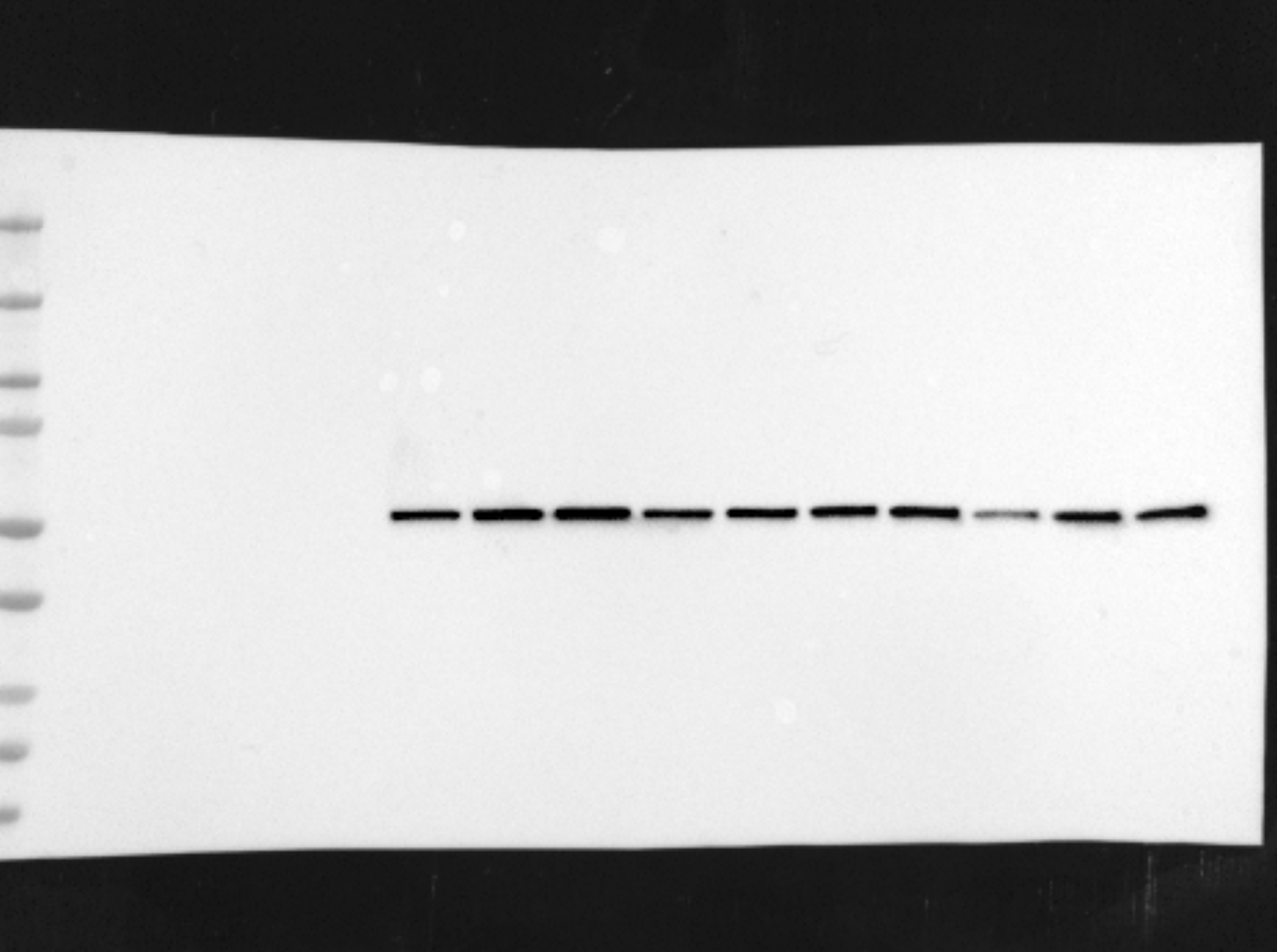

Supplement: Figure 5—source data 1. [file elife-69223-fig5-data1.zip › Figure 5/WesternBlots/Figure5B-2-full-raw-unedited.tif]

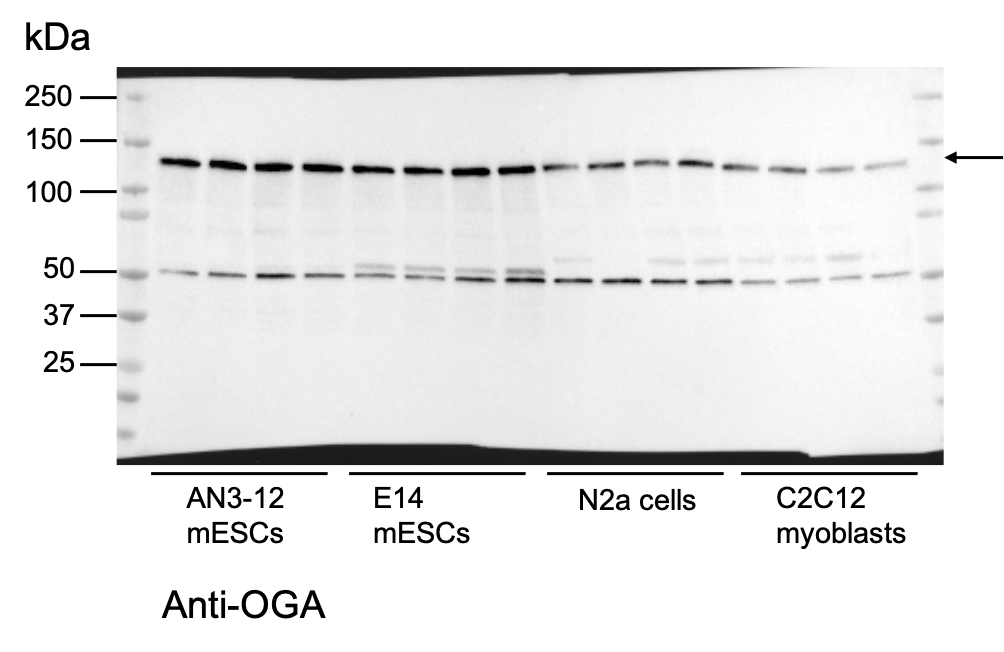

Supplement: Figure 5—source data 1. [file elife-69223-fig5-data1.zip › Figure 5/WesternBlots/Figure5G-2-labeled.png]

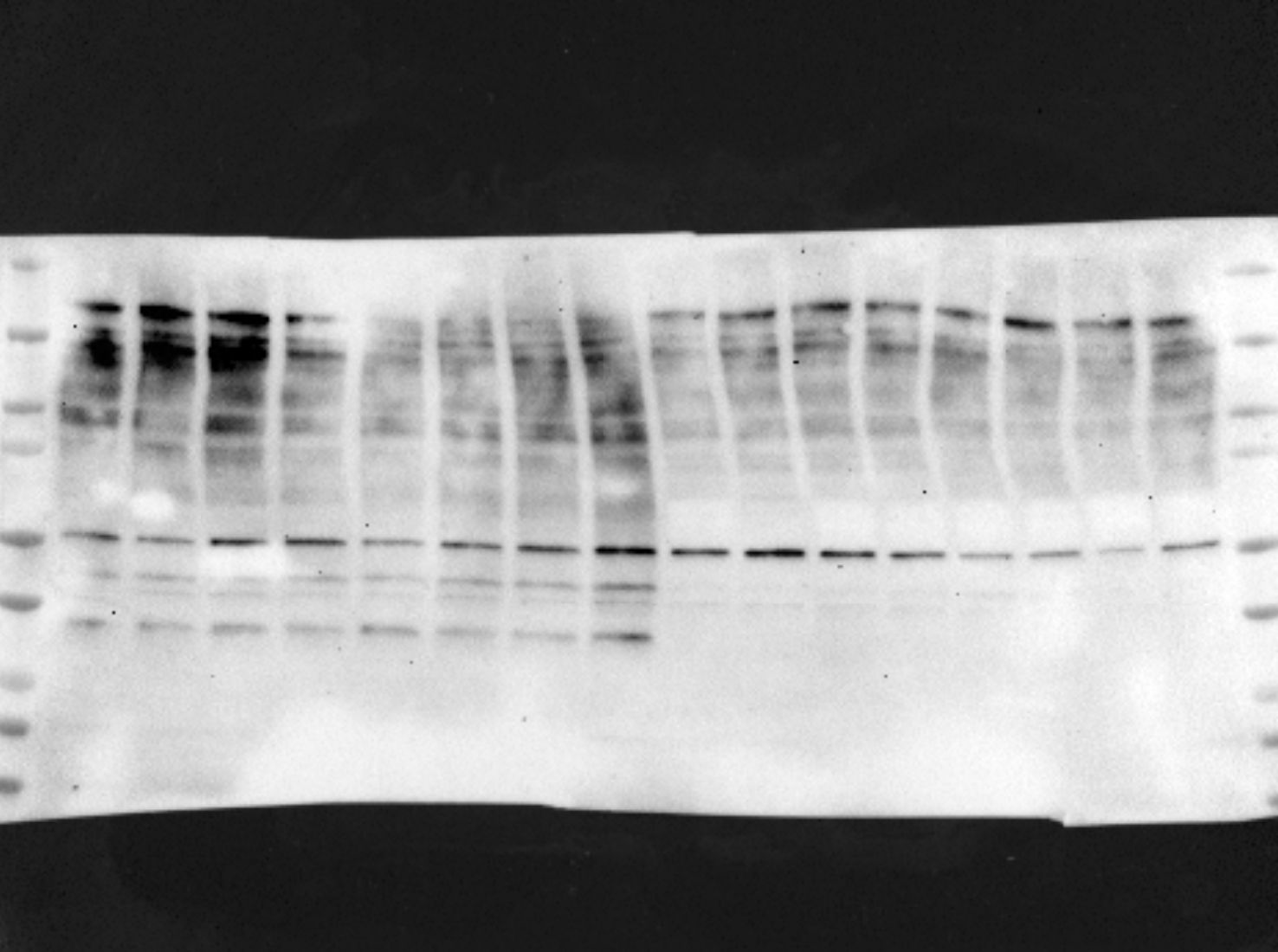

Supplement: Figure 5—source data 1. [file elife-69223-fig5-data1.zip › Figure 5/WesternBlots/Figure5G-1-full-raw-unedited.tif]

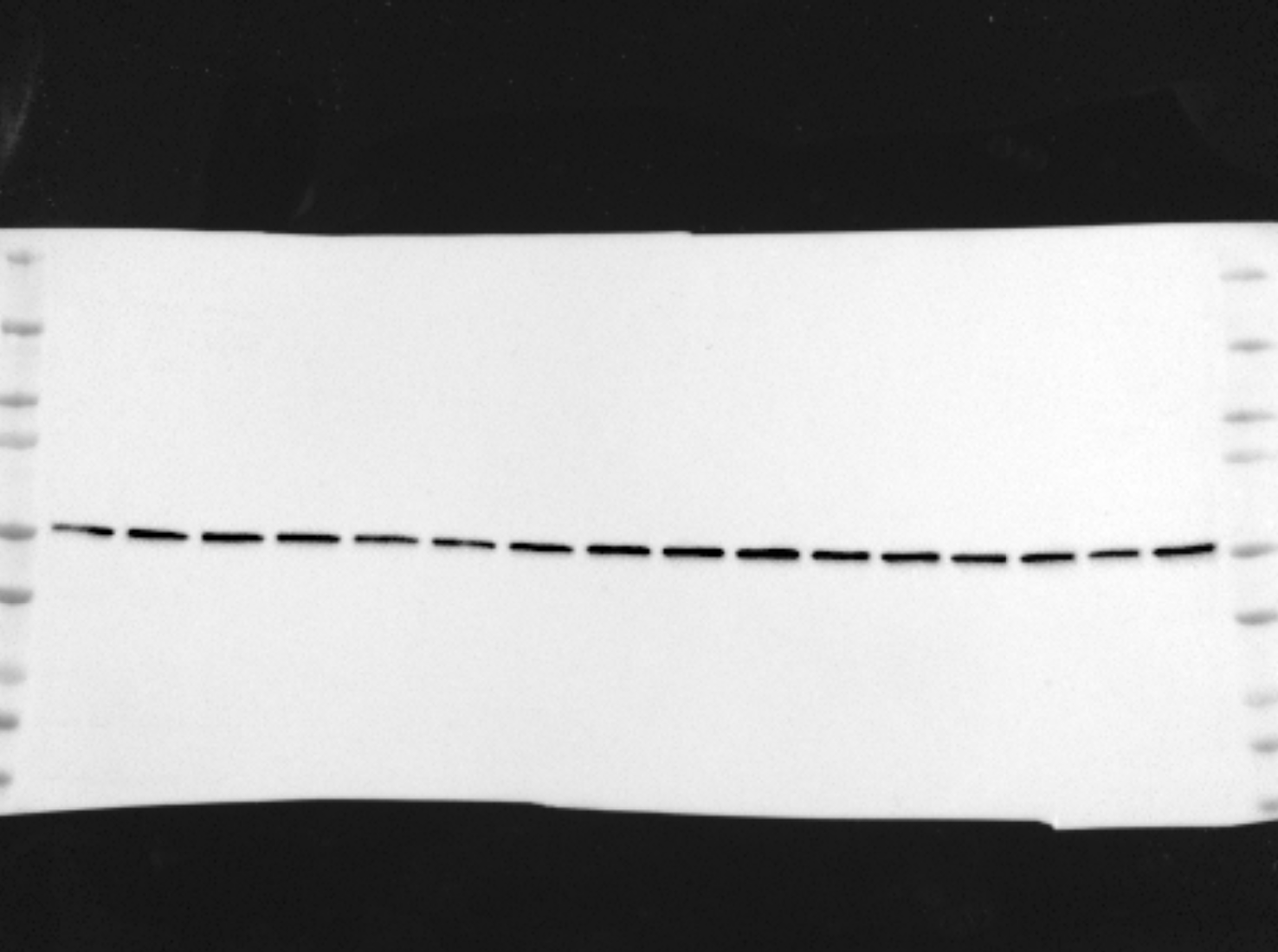

Supplement: Figure 5—source data 1. [file elife-69223-fig5-data1.zip › Figure 5/WesternBlots/Figure5G-4-full-raw-unedited.tif]

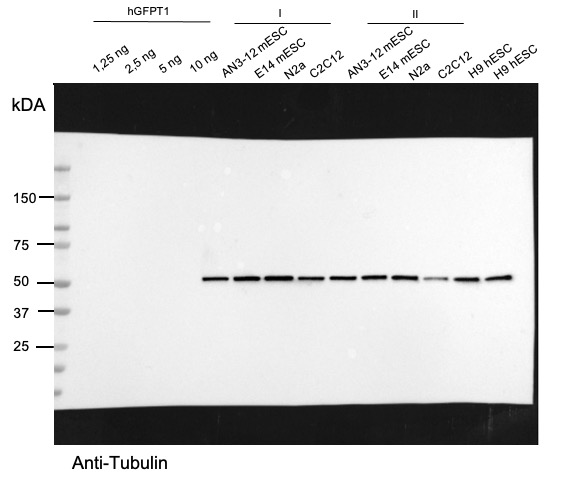

Supplement: Figure 5—source data 1. [file elife-69223-fig5-data1.zip › Figure 5/WesternBlots/Figure5B-2-labeled.jpg]

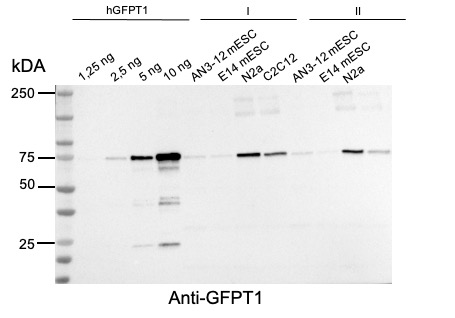

Supplement: Figure 5—source data 1. [file elife-69223-fig5-data1.zip › Figure 5/WesternBlots/Figure5B-1-labeled.jpg]

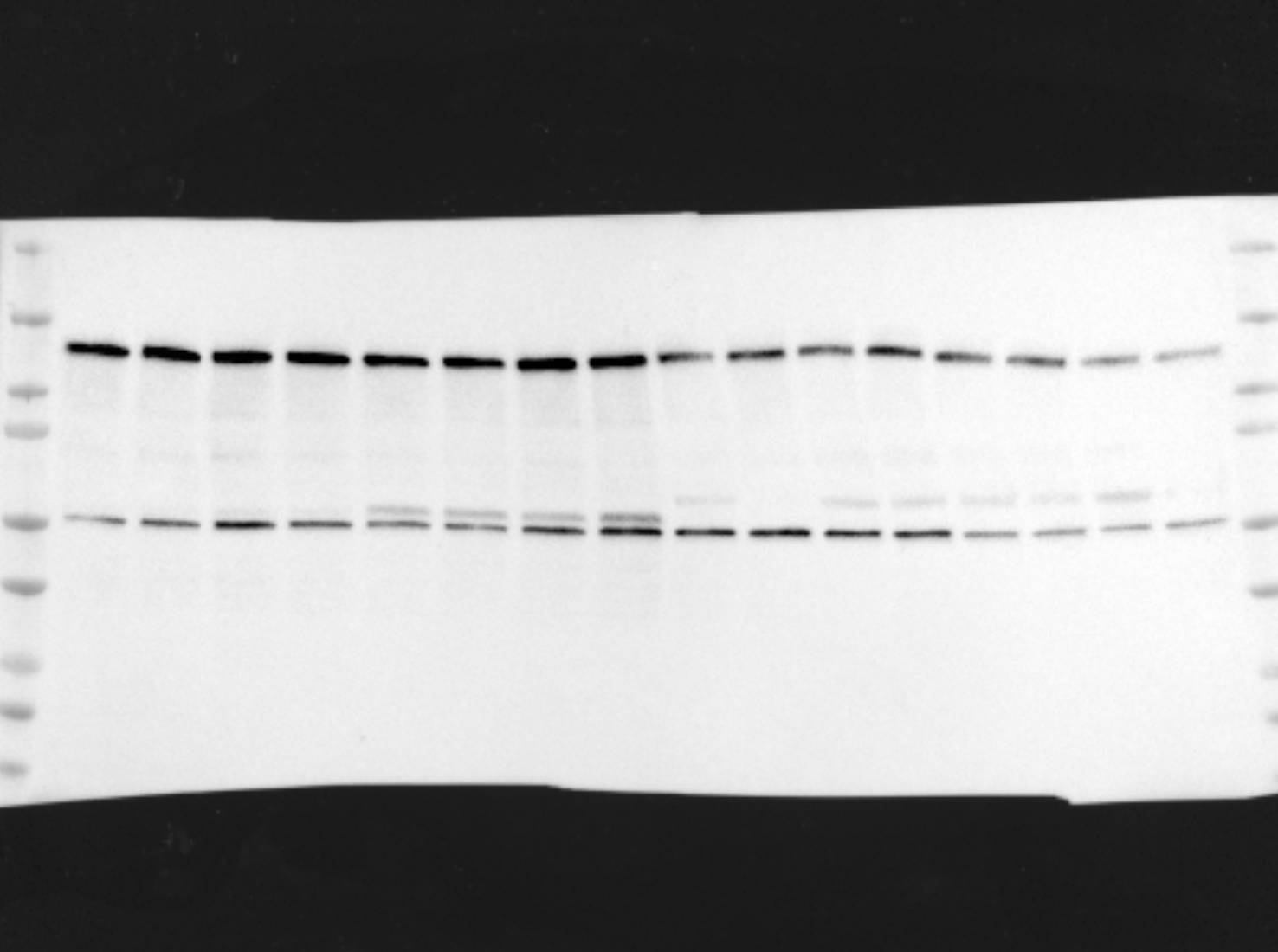

Supplement: Figure 5—source data 1. [file elife-69223-fig5-data1.zip › Figure 5/WesternBlots/Figure5G-2-full-raw-unedited.tif]

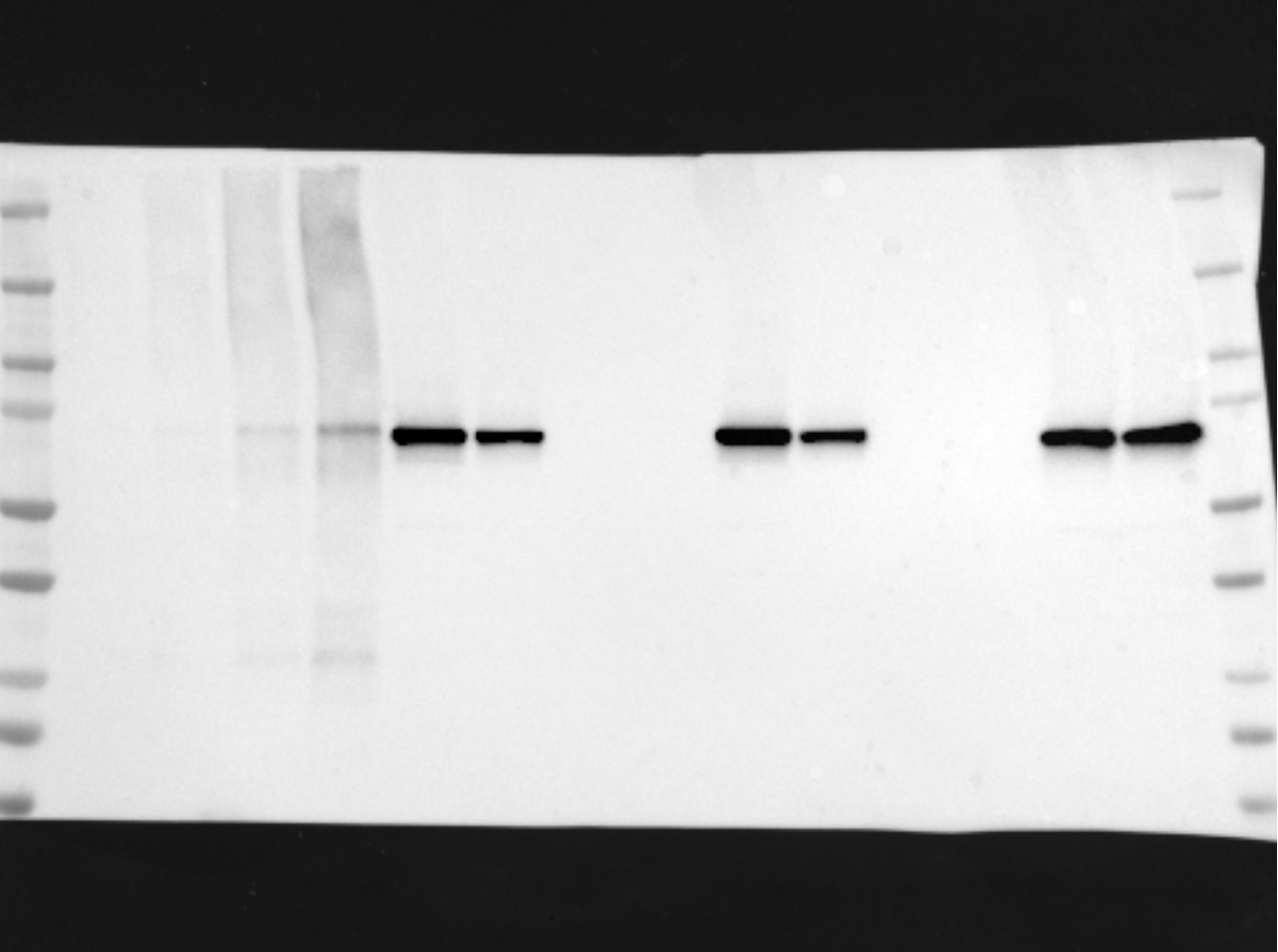

Supplement: Figure 5—source data 1. [file elife-69223-fig5-data1.zip › Figure 5/WesternBlots/Figure5B-3-full-raw-unedited.tif]

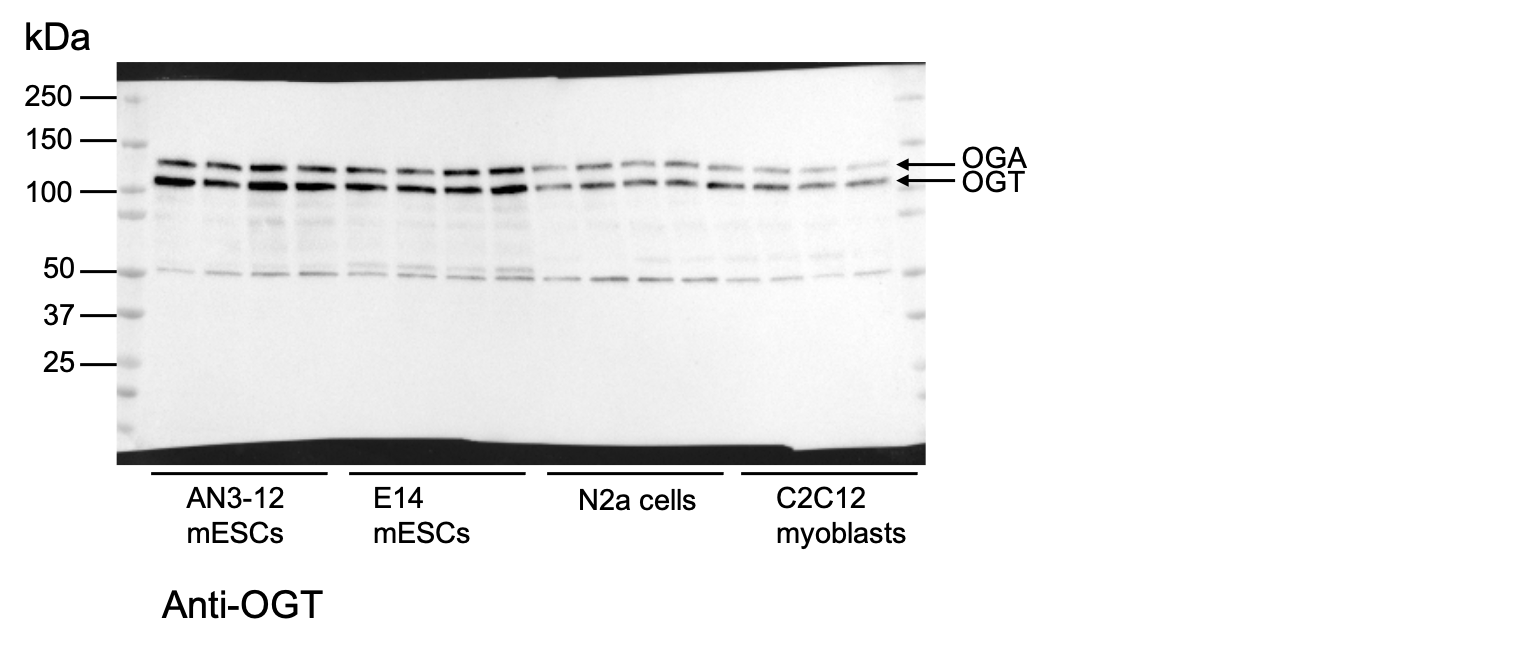

Supplement: Figure 5—source data 1. [file elife-69223-fig5-data1.zip › Figure 5/WesternBlots/Figure5G-3-labeled.png]

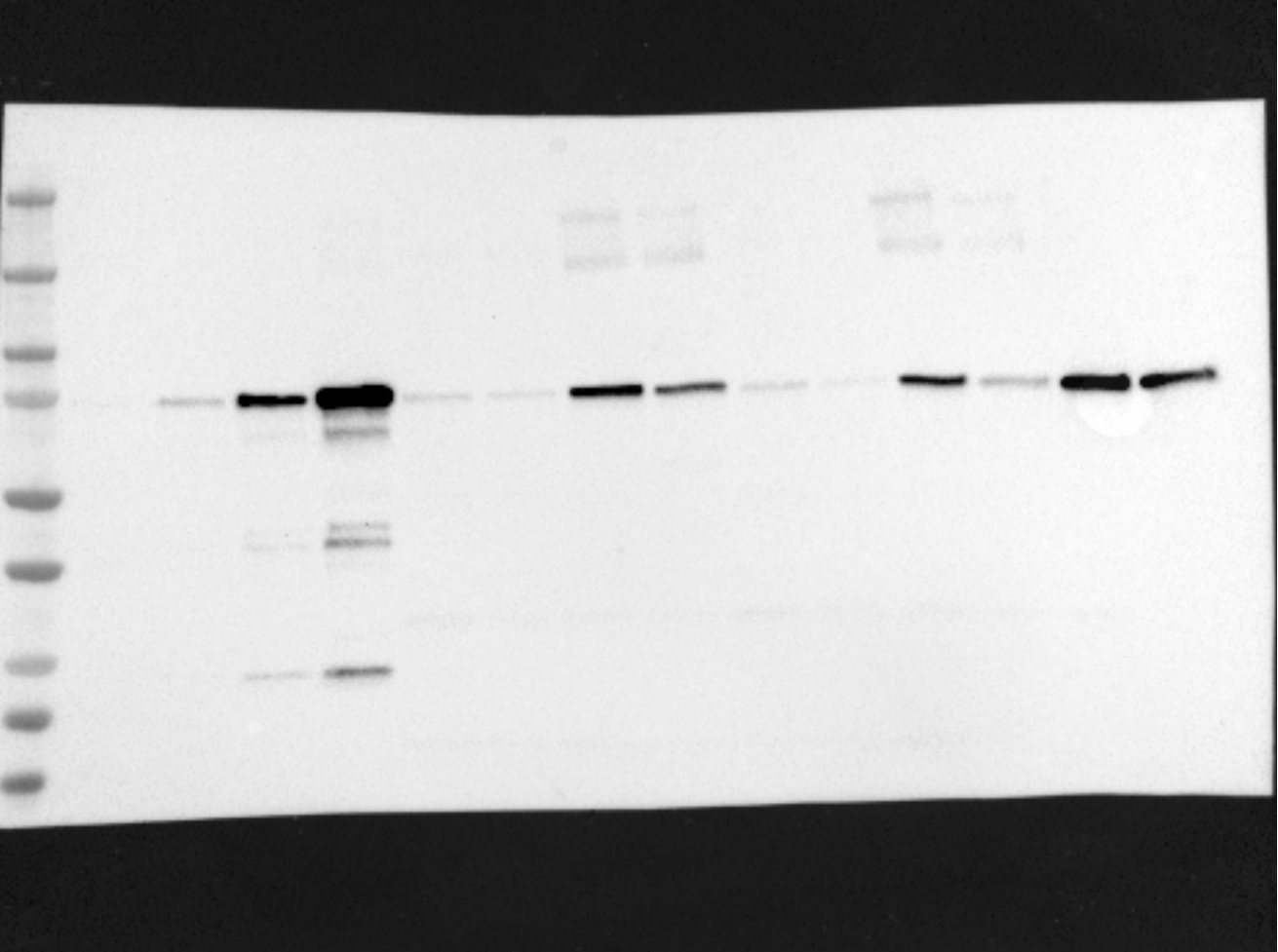

Supplement: Figure 5—source data 1. [file elife-69223-fig5-data1.zip › Figure 5/WesternBlots/Figure5B-1-full-raw-unedited.tif]

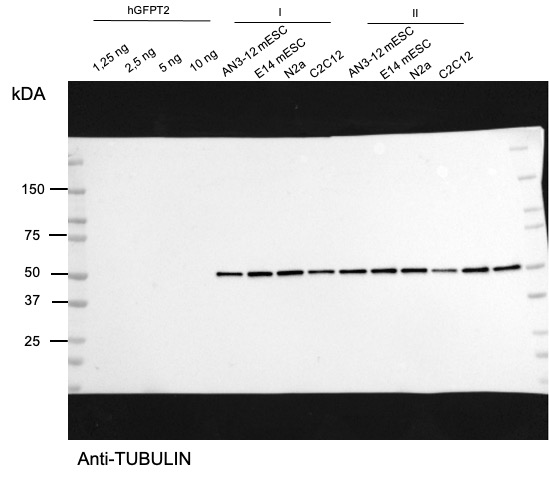

Supplement: Figure 5—source data 1. [file elife-69223-fig5-data1.zip › Figure 5/WesternBlots/Figure5B-4-labeled.jpg]

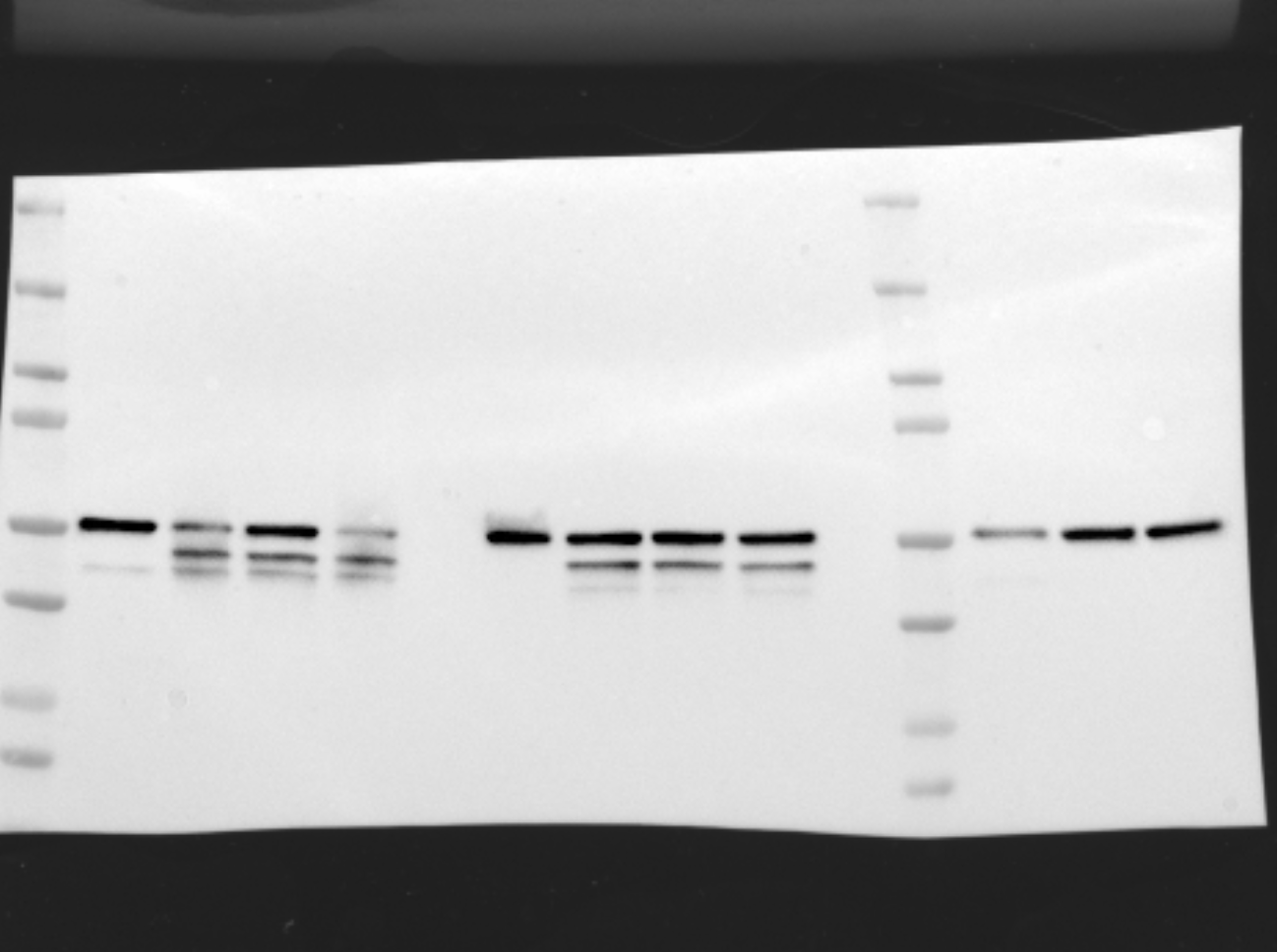

Supplement: Figure 5—figure supplement 2—source data 1. [file elife-69223-fig5-figsupp2-data1.zip › Figure 5-figure supplement 2/WesternBlots/Figure5FigureSupplement2a-2-full-raw-unedited.tif]

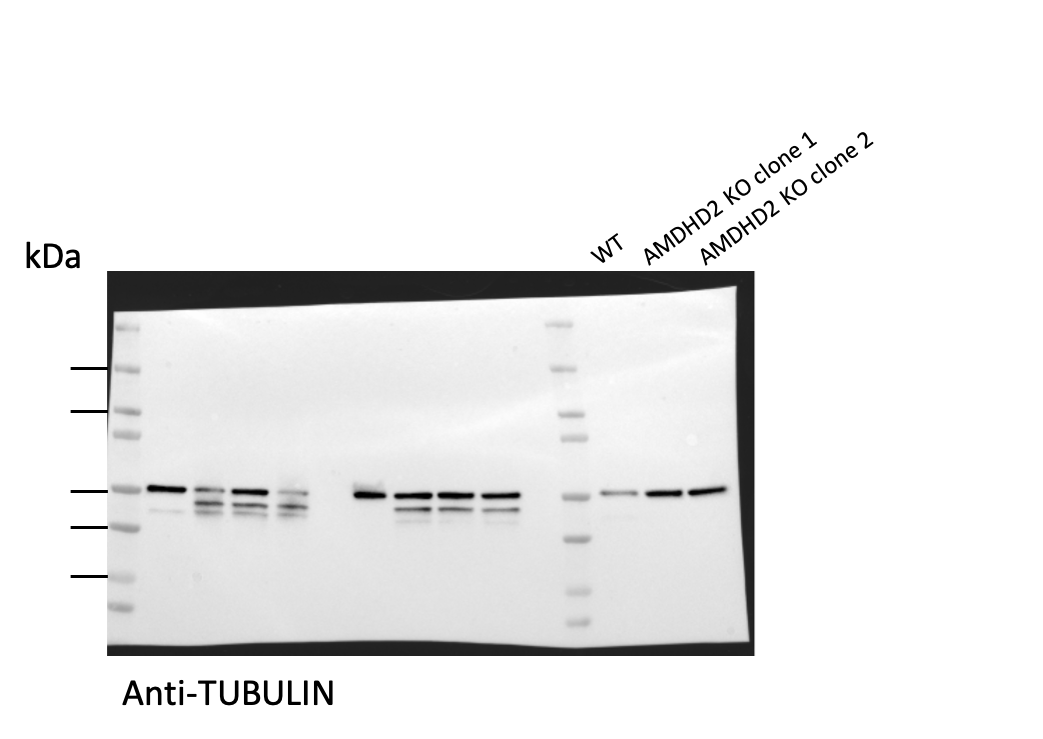

Supplement: Figure 5—figure supplement 2—source data 1. [file elife-69223-fig5-figsupp2-data1.zip › Figure 5-figure supplement 2/WesternBlots/Figure5FigureSupplement2a-2-labeled.png]

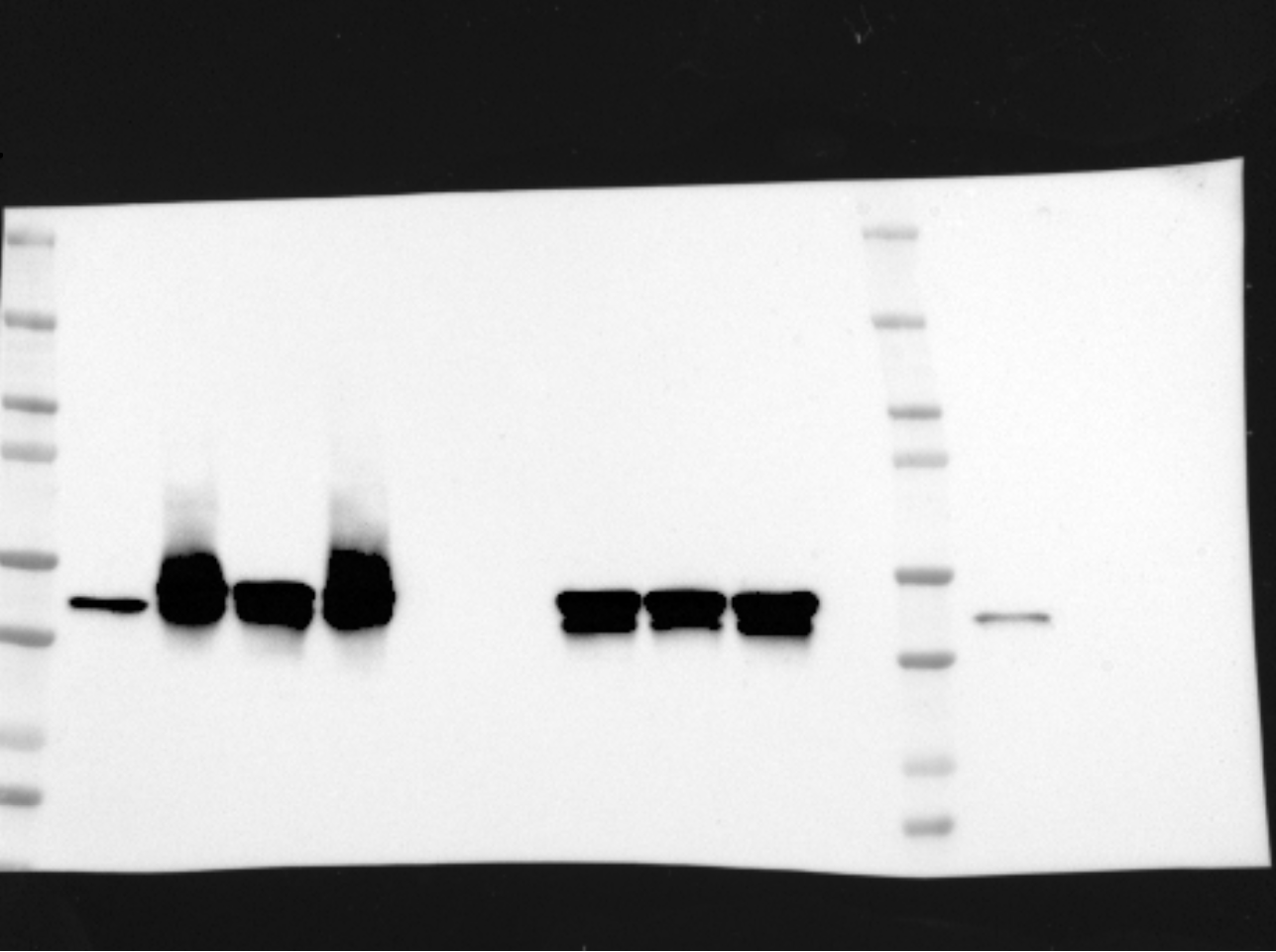

Supplement: Figure 5—figure supplement 2—source data 1. [file elife-69223-fig5-figsupp2-data1.zip › Figure 5-figure supplement 2/WesternBlots/Figure5FigureSupplement2a-1-full-raw-unedited.tif]

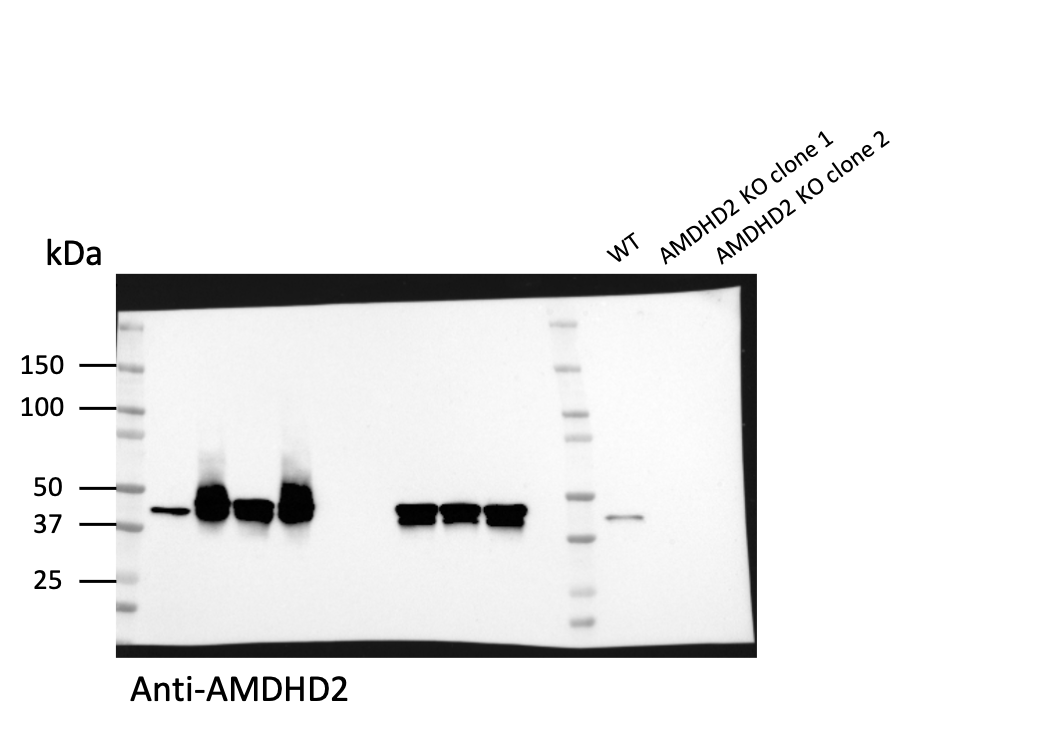

Supplement: Figure 5—figure supplement 2—source data 1. [file elife-69223-fig5-figsupp2-data1.zip › Figure 5-figure supplement 2/WesternBlots/Figure5FigureSupplement2a-1-labeled.png]

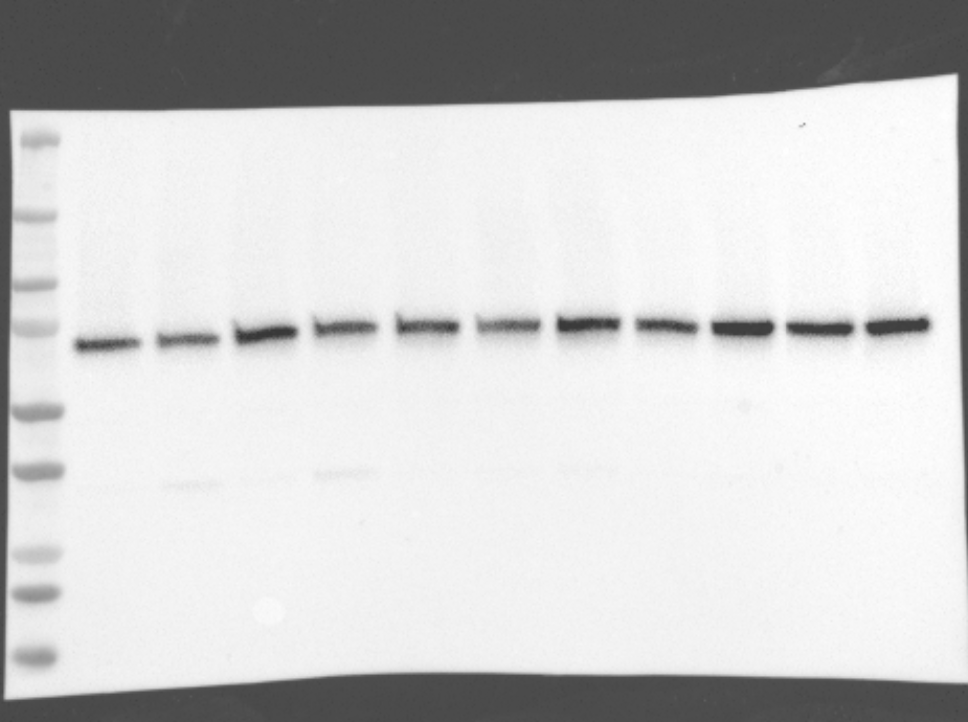

Supplement: Figure 6—source data 1. [file elife-69223-fig6-data1.zip › Figure 6/WesternBlots/Figure6A-1-full-raw-unedited.tif]

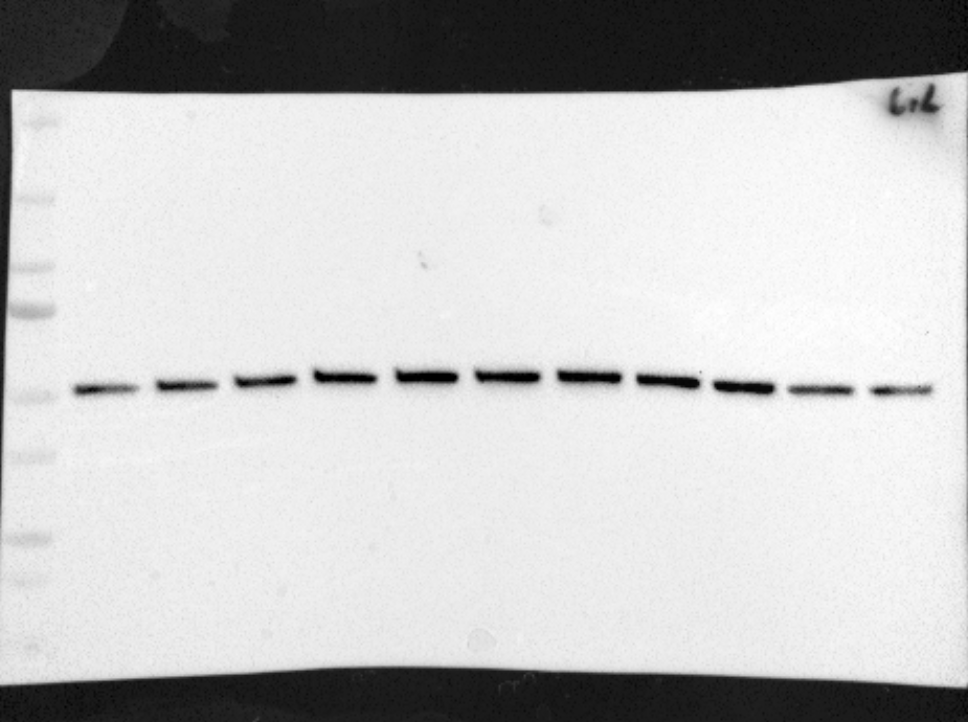

Supplement: Figure 6—source data 1. [file elife-69223-fig6-data1.zip › Figure 6/WesternBlots/Figure6A-2-full-raw-unedited.tif]

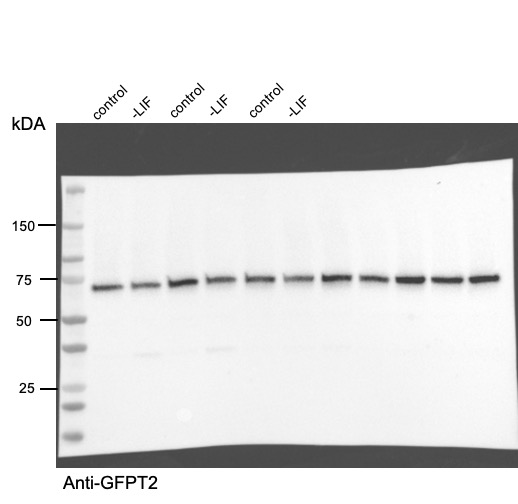

Supplement: Figure 6—source data 1. [file elife-69223-fig6-data1.zip › Figure 6/WesternBlots/Figure6A-1-labeled.jpg]

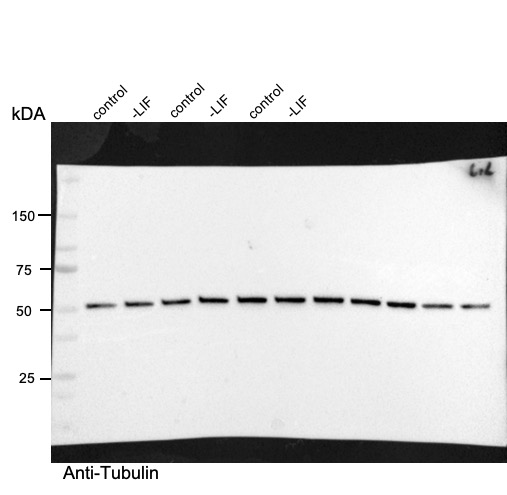

Supplement: Figure 6—source data 1. [file elife-69223-fig6-data1.zip › Figure 6/WesternBlots/Figure6A-2-labeled.jpg]

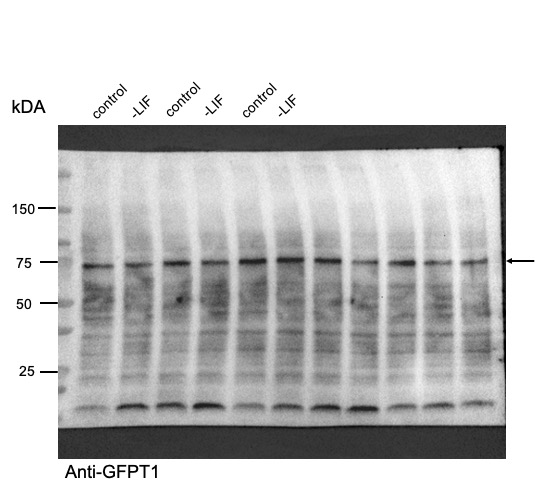

Supplement: Figure 6—figure supplement 1—source data 1. [file elife-69223-fig6-figsupp1-data1.zip › Figure 6-figure supplement 1/WesternBlots/Figure6FigureSupplement1C-1-labeled.jpg]

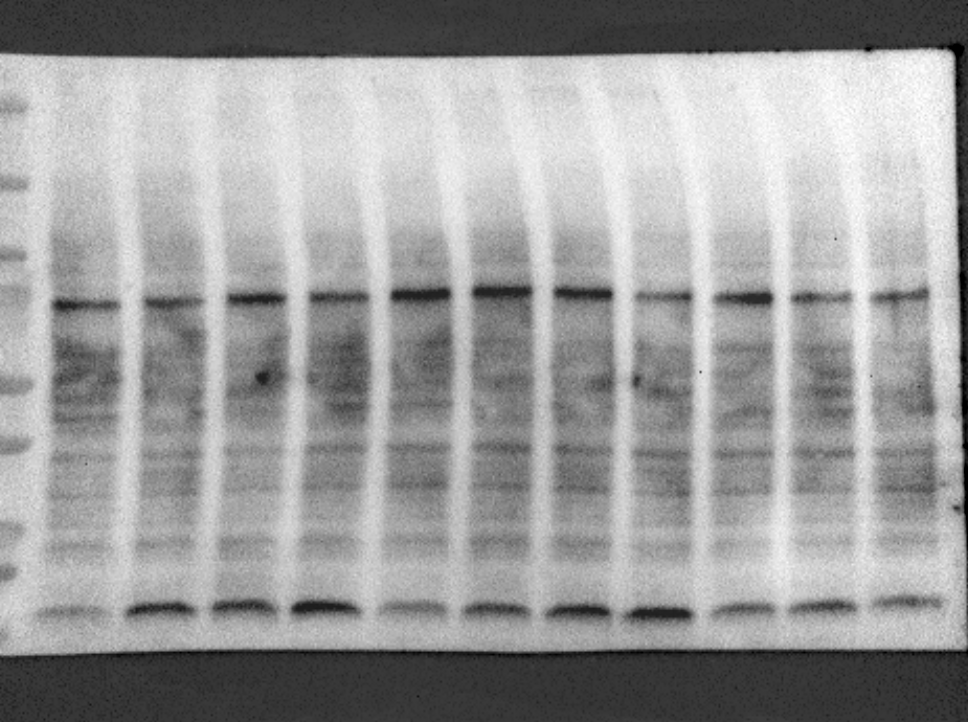

Supplement: Figure 6—figure supplement 1—source data 1. [file elife-69223-fig6-figsupp1-data1.zip › Figure 6-figure supplement 1/WesternBlots/Figure6FigureSupplement1C-1-full-raw-unedited.tif]

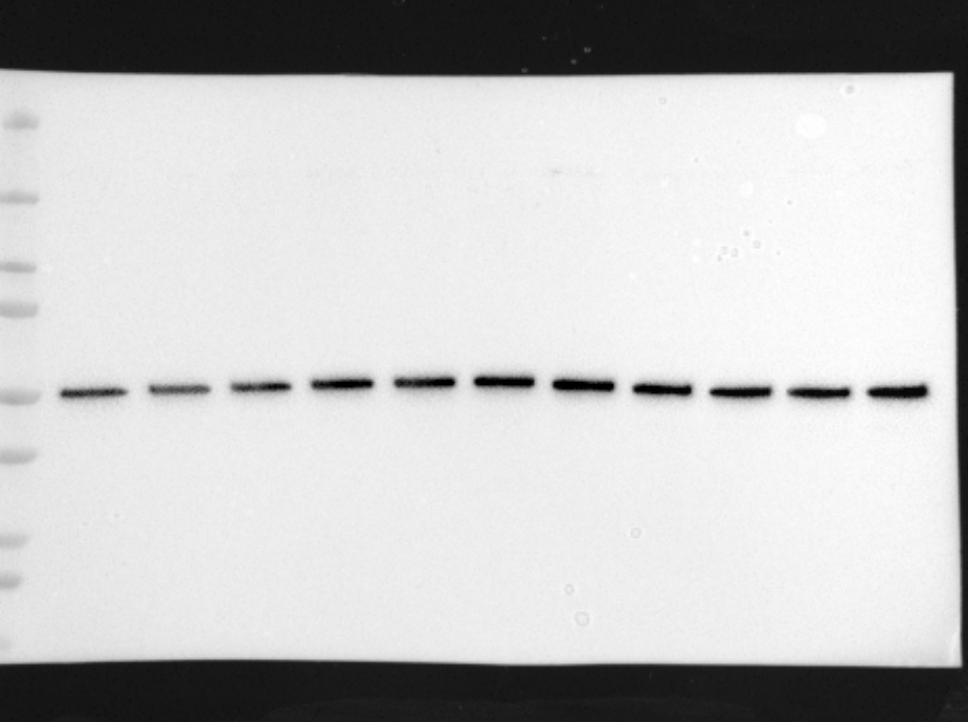

Supplement: Figure 6—figure supplement 1—source data 1. [file elife-69223-fig6-figsupp1-data1.zip › Figure 6-figure supplement 1/WesternBlots/Figure6FigureSupplement1C-3-full-raw-unedited.tif]

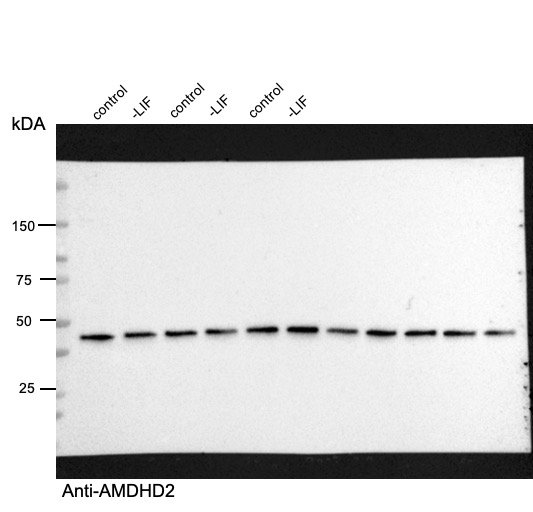

Supplement: Figure 6—figure supplement 1—source data 1. [file elife-69223-fig6-figsupp1-data1.zip › Figure 6-figure supplement 1/WesternBlots/Figure6FigureSupplement1C-2-labeled.jpg]

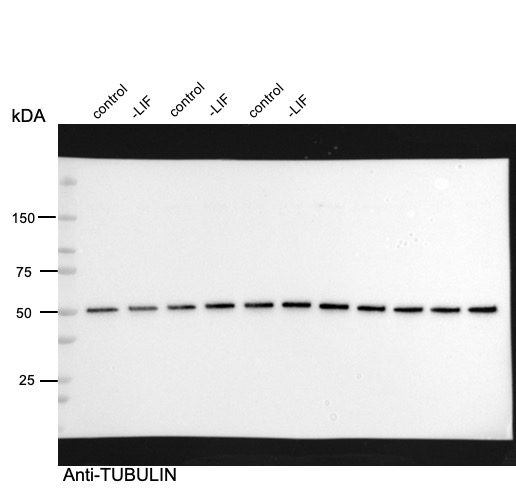

Supplement: Figure 6—figure supplement 1—source data 1. [file elife-69223-fig6-figsupp1-data1.zip › Figure 6-figure supplement 1/WesternBlots/Figure6FigureSupplement1C-3-labeled.jpg]

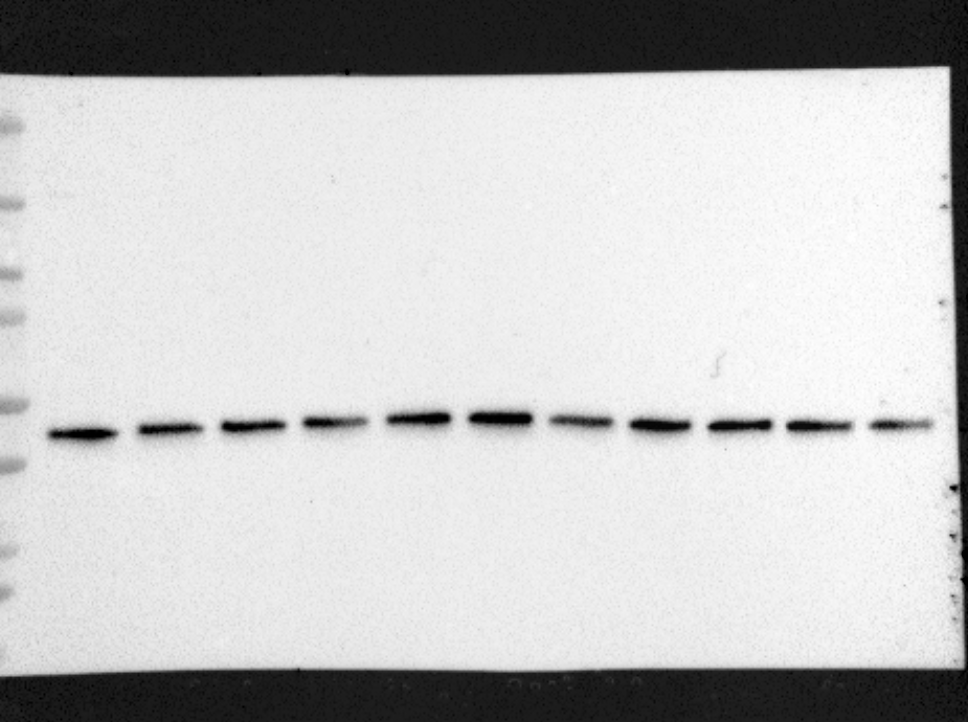

Supplement: Figure 6—figure supplement 1—source data 1. [file elife-69223-fig6-figsupp1-data1.zip › Figure 6-figure supplement 1/WesternBlots/Figure6FigureSupplement1C-2-full-raw-unedited.tif]
