# Supplementary material for: GFPT2/GFAT2 and AMDHD2 act in tandem to control the hexosamine pathway
Source: eLife. 2022 Mar 1;11:e69223. doi: 10.7554/eLife.69223 (PMC8970586; doi:10.7554/eLife.69223)
Supplement: Supplementary file 1. [file elife-69223-supp1.docx]

**Supplementary Table 1 | Primers used in this study.**

| Primer ID | Sequence (5’ à 3’) | Purpose |
| --- | --- | --- |
| hAMDHD2_I38T_for | ggccgcaCTttggacccagagaagc | site-directed mutagenesis |
| hAMDHD2_I38T_rev | gggtccaaagtgcggcctccgcgca | site-directed mutagenesis |
| hAMDHD2_G102D_for | tcgcacgAcgtcacctccttctgcc | site-directed mutagenesis |
| hAMDHD2_G102D_rev | gaggtgacgtcgtgcgacaggatcc | site-directed mutagenesis |
| hAMDHD2_G130R_for | aagagtCGtggtccccatggggcag | site-directed mutagenesis |
| hAMDHD2_G130R_rev | tggggaccacgactcttcacaggga | site-directed mutagenesis |
| hAMDHD2_L142F_for | ctgcacTtCgagggccccttcatca | site-directed mutagenesis |
| hAMDHD2_L142F_rev | gggccctcgaagtgcagcccgagga | site-directed mutagenesis |
| hAMDHD2_F146L_for | ggccccttGatcagccgggagaagc | site-directed mutagenesis |
| hAMDHD2_F146L_rev | cggctgatcaaggggccctccaggt | site-directed mutagenesis |
| hAMDHD2_A154P_for | gggcCcAcaccccgaggcccacct | site-directed mutagenesis |
| hAMDHD2_A154P_rev | tcggggtgtgggccccgcttctccc | site-directed mutagenesis |
| hAMDHD2_T185A_for | atcgtgGcTctggccccagagttgg | site-directed mutagenesis |
| hAMDHD2_T185A_rev | ggggccagagccacgatgcggacat | site-directed mutagenesis |
| hAMDHD2_S208T_for | tgcgtgAcTctagggcactcagtgg | site-directed mutagenesis |
| hAMDHD2_S208T_rev | tgccctagagtcacgcagatgccac | site-directed mutagenesis |
| hAMDHD2_G226E_for | tggagcgAagccaccttcatcaccc | site-directed mutagenesis |
| hAMDHD2_G226E_rev | aaggtggcttcgctccacacagcat | site-directed mutagenesis |
| hAMDHD2_H242A,R243A_for | cctttccacGCcGCcgacccaggcatcgtg | site-directed mutagenesis |
| hAMDHD2_H242A,R243A_rev | gggtcgGCgGCgtggaaaggcagcatggc | site-directed mutagenesis |
| hAMDHD2_G265V_for | ttctatgTCatgattgcagatggcacgc | site-directed mutagenesis |
| hAMDHD2_G265V_rev | gcaatcatgacatagaagatgcagcggc | site-directed mutagenesis |
| hAMDHD2_G265R_for | ttctatCgTatgattgcagatggcacgc | site-directed mutagenesis |
| hAMDHD2_G265R_rev | gcaatcatacgatagaagatgcagcggc | site-directed mutagenesis |
| hAMDHD2_I280E_for | cctgcggGAAgcccaccgtgcccatc | site-directed mutagenesis |
| hAMDHD2_I280E_rev | gtgggcTTCccgcagggcggcggg | site-directed mutagenesis |
| hAMDHD2_I280R_for | ctgcggCGTgcccaccgtgcccatc | site-directed mutagenesis |
| hAMDHD2_I280R_rev | gggcACGccgcagggcggcggg | site-directed mutagenesis |
| hAMDHD2_D294A_for | gtcaccgCtgccatccctgcc | site-directed mutagenesis |
| hAMDHD2_D294A_rev | ggcaGcggtgaccagcaccag | site-directed mutagenesis |
| hGFAT2-300-His6_SDM_for | cgctcggccagtCATCACCATCACCATCACgatgacccatctcgagccatccagaccttgcagatggaac | integration of internal His_6_tag in GFAT-2 |
| mGFAT1_G451E_guide 1 | GAGTCGGCAGTTCTATATCA | CRISPR/Cas9 |
| mGFAT1_G451E_guide 2 | GGTGGGGATCACAAATACAGT | CRISPR/Cas9 |
| mAMDHD2_guide1 | GTTCATCAGCCGGGAAAAGCG | CRISPR/Cas9 |
| mAMDHD2_guide2 | GCATTGGCTTCAAAGGAGCGG | CRISPR/Cas9 |
| mAMDHD2_guide3 | GAGGAGCGGAGGTAGGCCTCG | CRISPR/Cas9 |
| mAMDHD2_guide4 | GCTTTTCCCGGCTGATGAAT | CRISPR/Cas9 |
| mAMDHD2_guide5 | GCCCTCCAAGTGCACCCCTGA | CRISPR/Cas9 |
| mGFAT1_G451E_repair template | GGCGAGACAGCTGACACCCTGATGGGACTTCGTTACTGTAAGGAGAGAGGAGCCTTAACTGTGGGCATCACTAATACAGTCGAAAGTTCCATATCAAGAGAGACAGATTGCGGGGTTCATATTAATGCTGGTCCTGAGATTGGCGTGGCCAGTACAAAG | CRISPR/Cas9 |
| mGFAT1_G451E_geno_for | AGTCGGTTGGTTTTTCGTGT | Genotyping |
| mGFAT1_G451E_geno_rev | ACTGCCCCACAGATCAGAGT | Genotyping |
| mAMDHD2_geno_for | GGCCTTCATCTTCAGCTCCT | Genotyping |
| mAMDHD2_geno_rev | TGAGATCAGTTTCTGCAGCAG | Genotyping |
| DS | GAGCCAGAACCAGAAGGAACTTGAC | Genotyping |
| US | GTGACTGGAGTTCAGACGTGTGCTCTTC | Genotyping |
| mAMDHD2_geno_for  (transgenic mice) | TCTAGCTGTTTGGCCAAAGC | Genotyping |
| mAMDHD2_geno_rev  (transgenic mice) | AGAGACACACGGATGCCTTG | Genotyping |
| *mGfat1*_qPCR_for | AAAGGAAGCTGCGGTCTTTCCC | qPCR |
| *mGfat1*_qPCR_rev | GTGTGCTCTATCACGGCACTTG | qPCR |
| *mGfat2*_qPCR_for | CTACAGAACAGGAGACAAGAGATC | qPCR |
| *mGfat2*_qPCR_rev | GTTATATCCCCGTCCCATCAC | qPCR |
| *mAmdhd2*_qPCR_for | CTTGCGCCCAAAGCTTG | qPCR |
| *mAmdhd2*_qPCR_rev | ACCCACAGATCCTCCCTG | qPCR |
| *mNanong*_qPCR_for | ATGAAGTGCAAGCGGTGGCAGAAA | qPCR |
| *mNanong*_qPCR_rev | CCTGGTGGAGTCACAGAGTAGTTC | qPCR |
| *mKlf4*_qPCR_for | ACAGGCGAGAAACCTTACCACTGT | qPCR |
| *mKlf4*_qPCR_rev | GCCTCTTCATGTGTAAGGCAAGGT | qPCR |
| *mGapdh*_qPCR_for | GGCATGGACTGTGGTCATGAG | qPCR |
| *mGapdh*_qPCR_rev | TGCACCACCAACTGCTTAGC | qPCR |
